# B cell phenotypes and antibody signatures associate with interpatient variation in the lung adenocarcinoma tumor microenvironment

**DOI:** 10.3389/fimmu.2025.1739637

**Published:** 2026-01-28

**Authors:** Kamille M. Rasche, David Tieri, Samantha M. Morrissey, Hong Li, Fan Zhang, William S. Gibson, Easton E. Ford, Uddalok Jana, Melissa L. Smith, Jun Yan, Corey T. Watson

**Affiliations:** 1 Department of Microbiology and Immunology, University of Louisville School of Medicine, Louisville, KY, United States; 2 Department of Biochemistry and Molecular Genetics, University of Louisville School of Medicine, Louisville, KY, United States; 3 Department of Medicine, University of Louisville School of Medicine, Louisville, KY, United States; 4 Department of Surgery, University of Louisville School of Medicine, Louisville, KY, United States

**Keywords:** antibody repertoire, B cells, cytometry by time-of-flight, lung adenocarcinoma, sequencing

## Abstract

**Introduction:**

Non-small cell lung cancer is the leading cause of cancer-related mortality worldwide, with lung adenocarcinoma (LUAD) as the most common subtype. Although early-stage disease is often treated surgically, advanced LUAD typically requires chemotherapy, radiation, and/or immunotherapy, largely focused on T cell–mediated responses. Therapeutic efficacy, however, is also shaped by tumor-infiltrating (TI) B cells, whose roles in LUAD remain incompletely understood.

**Methods:**

We performed cytometry by time of flight (CyTOF) using 44 markers on matched tumor, adjacent lung, and peripheral blood samples from 48 LUAD patients to define TI immune landscapes. 66 immune cell subsets were identified, and patients were stratified into four groups based on TI cell composition. Adaptive immune receptor repertoire sequencing (AIRR-seq) of IgM and IgG was conducted on matched samples from 29 patients to assess clonal expansion and affinity maturation. Subisotype-resolved AIRR-seq was additionally performed on tumor samples from 18 patients.

**Results:**

CyTOF analysis revealed four patient groups with distinct TI immune profiles. AIRR-seq demonstrated increased clonal expansion and affinity maturation in tumors compared to adjacent lung and blood. Tumor-specific IGHV enrichment patterns were observed but were not associated with patient group assignment. Instead, clonal expansion was greatest in tumors with higher lymphocyte proportions. Subisotype-resolved analysis showed enrichment of IGHG2 and IGHG3 in tumors from patients with low TI B cell abundance, whereas IGHG4 was enriched in patients with high TI B cell infiltration and correlated with four CyTOF-defined immune subsets.

**Discussion:**

These findings reveal substantial inter-individual variation in TI immune landscapes and highlight distinct B cell repertoire and subisotype features within LUAD tumors. Together, these data suggest that B cell composition and antibody subisotype usage may contribute to immune contexture and could inform the development of more tailored immunotherapeutic strategies in LUAD.

## 1 Introduction

Non-small cell lung cancer (NSCLC) is the leading cause of cancer-related deaths, with lung adenocarcinoma (LUAD) as the most common subtype (1). Early-stage LUAD can often be treated successfully with surgery, but advanced stages typically require chemotherapy, radiation, and/or immunotherapy (2, 3). Immunotherapies mainly target T cells, but responses vary, likely due to the complexity of the tumor microenvironment (TME) (1–3). In LUAD, varying proportions of tumor-infiltrating (TI) immune cells, such as PD-1+ T cells, dendritic cells (DCs), and B cells, have been linked to different clinical outcomes, with TI B cells in particular showing potential as modulators of beneficial anti-tumor immune responses (3–5).

Studies have shown B cells elevated in the tumors of some LUAD patients compared to adjacent tissues, and high densities of B cells in tertiary lymphoid structures (TLS) have been consistently associated with a better prognosis (5–9). TI B cells have been shown to influence T cell activity and may also independently affect tumor progression, yet the diversity and specific functions of B cell subsets remain unclear (8, 10–13). Previous studies in other solid cancers have shown B cells perform additional roles, including direct tumor cell killing and enhancement of CD8+ T cell activity via CD27 signaling, though these functions have not been fully explored in LUAD (14–17).

Antibodies, the secreted form of B cell receptors (BCRs) are key factors in immune responses, particularly in cancer (7, 10, 12, 15). Tumor-associated IgG and IgA subisotypes have been linked to favorable prognosis due to their ability to mediate antibody-dependent cytotoxicity (ADCC) and phagocytosis (ADCP) (15, 18). Supporting the presence of functional antibodies in TME, Germain et al. (2014) detected secreted IgG and IgA in LUAD tumor supernatants that bound common tumor antigens (19). IgG1, for instance, has been repeatedly linked to a positive prognosis in solid cancers due to its ability to induce phagocytosis and cytotoxicity through Fc receptor binding on macrophages and natural killer (NK) cells (7, 20). In a single LUAD study, high IgG1 transcript expression in tumors was associated with favorable outcomes in patients with KRAS mutations, while elevated IgG4 expression was linked to better outcomes in patients with STK11 mutations (21). Despite these findings, our understanding of IgG subisotype distributions in the TME and the potential effects on TI cells remain limited (20, 21).

Some studies have investigated BCR diversity in LUAD using adaptive immune receptor repertoire sequencing (AIRR-seq) to assess the diversity and clonal expansion of B cells (10, 12, 13, 22). These have revealed biased gene usage and clonal expansion in LUAD, identifying variable (V) and joining (J) gene combinations linked to prognosis and recurrence (23, 24). Other studies have highlighted increased somatic hypermutation (SHM) in tumors, further emphasizing the significance of BCR diversity in shaping immune responses and influencing clinical outcomes (12, 18). However, these studies lack resolution and context of the cellular environment, limiting insight into how BCR repertoire features relate to specific immune cells within the TME. Moreover, isotype-sepecific patterns in V, diversity (D), and J gene usage and SHM remain largely unexplored.

Here, we investigated the TME of LUAD patients, focusing on characterizing inter-tissue and inter-individual variation in B cell, T cell, and myeloid populartions. Using cytometry by time of flight (CyTOF) with a 44–immune cell surface marker panel across matched tissues from 48 LUAD patients, we identified novel TI cell subset proportions and combinations that could be used to define groups of patients. By examining the surface expression of cell type–specific lineage and functional markers, we discerned phenotypes and inferred functions of 66 immune cell subsets, thereby expanding our understanding of distinct phenotypes that were elevated within the tumor. AIRR-seq highlighted clonal expansion in tumor tissues, especially in patients with high TI lymphocyte levels. Distinct changes in heavy chain V, D, and J gene segment usage across tissues further highlighted the secretion of tumor-associated BCRs. Additionally, we explored the associations between distinct immune cell profiles and IgG subisotypes using a novel near full-length adaptive immune receptor repertoire sequencing method (FLAIRR-seq). Together, these analyses refined our understanding of inter-individual variation in TI immune profiles, establishing a foundation for investigating the role of this variation in disease prognosis, therapeutic approaches, and treatment outcomes.

## 2 Methods

### 2.1 Sample collection and processing

Fresh tumor, adjacent non-tumor lung tissue, and PBMC were obtained from early-stage LUAD patients (n = 49) undergoing surgical resection at the James Graham Brown Cancer Center, University of Louisville. Patients were treatment naive or, in the case of three patients, had not received chemo/radiation treatment at least 65 days prior to surgery. Samples were anonymously coded in accordance with the Declaration of Helsinki; written informed consent was obtained under protocols approved by the University of Louisville School of Medicine IRB. Cells were isolated, counted, resuspended in FBS/10% DMSO, and stored at –150°C.

### 2.2 Flow cytometry

Fresh tumor and adjacent tissues (n = 12) were minced and digested in RPMI 1640 with 2% FBS, type IV collagenase (1 µg/ml), and hyaluronidase (10 ng/ml) for 20–40 min at 37°C. Single-cell suspensions were stained with fluorescently labeled mAbs (CD45, CD19, CD69, CD27, CD38, viability dye) and analyzed on a BD Canto II flow cytometer.

### 2.3 Immunofluorescent staining

Human lung cancer and distant tissues were snap-frozen in OCT and stored at –80°C. Eight-µm sections were fixed with cold acetone for 15 min and air-dried for 30 min. Slides were blocked with 20% FBS in PBS for 1 h, stained overnight at 4°C with anti-CD20-PE (1:100), CD3-FITC (1:100), and DAPI, and imaged on a Nikon A1R confocal microscope.

### 2.4 CyTOF sample preparation and data acquisition

Preserved cells were thawed quickly in a 37°C water bath and washed three times in pre-warmed complete RPMI 1640 media and then counted to determine proper input. Cell aliquots were then resuspended in serum free RPMI and incubated with Cisplatin (Fluidigm; Cat#201064, 1:1000) for 5 minutes at room temperature. After incubation, cells were washed with 5X volume of pre-warmed complete RPMI 1640, then re-suspended and washed in 4mL Maxpar Cell Staining Buffer (Fluidigm, Cat#201068). Cells were incubated for 10 minutes at room temperature with 2uL of Human TruStain FcX (Biolegend) and stained with a panel of 44 CyTOF antibodies for 30 minutes. CyTOF antibodies were either purchased pre-conjugated (Fluidigm) or conjugated in house using either Maxpar MCP9 antibody labeling kits or Maxpar X8 antibody labeling kits (**Supplementary Table 1**). Cells were washed with Maxper Cell Staining Buffer and fixed with 1.6% Formaldehyde (Thermofisher, Cat#28906) in Maxpar PBS (Fluidigm, Cat#201058) for 10 minutes at room temperature. Then cells were re-suspended with 1mL of Cell-ID™ Intercalator-Ir—125 µM at 1:8000 in Maxpar Fix and Perm Buffer (Fluidigm, Cat#201067) and incubated at 4°C. The following morning, cells were washed with 2mL Maxpar Cell Staining Buffer once and 1mL of Maxpar Cell Acquisition solution (Fluidigm, Cat#201241) once. Pelleted cells were kept on ice until analysis. Cells were then resuspended in EQ Four Element Calibration Beads (Fluidigm, Cat#201078)/Maxpar Cell Acquisition solution at 1:10 ratio with cell concentration around 1.0x10^6^ cells/mL. Cells were filtered before acquisition on a Helios (Fluidigm) at approximately 500 events/second. Standard gating analysis identifying the number of live immune cells (CD45+; mean=82,226 cells per patient), T cells (CD3+CD4+, CD3+CD8+; mean=44,616 cells per patient) and B cells (CD19+; mean=9,344 cells per patient) was conducted through FlowJo software (BD Life Sciences) for subsequent clustering and differential analysis.

### 2.5 CyTOF data processing and analysis

CyTOF data was analyzed using the CyTOF Workflow (25, 26). Briefly, FlowJo workspace files were imported and parsed using functions from flowWorkspace and CytoML, then stored as a single cell experiment object after arcsinh transformation through CATALYST (25, 27). Unsupervised cell clustering was conducted using FlowSOM and ConsensusClusterPlus in two parts (25, 26). Initial clustering was done by first compiling all events from each sample and using primary lineage markers CD19, CD3, CD4, CD8, and CD11b to generate 20 unique clusters. Clusters were then labeled as CD19+ B cells, CD3+CD4+ T cells, CD3+CD8+ T cells, or CD11b+ myeloid cells and merged for use in secondary clustering. CD56+CD16+ clusters or CD3+TCRgd+ clusters were not merged and labeled NK or TCRgd T cells, respectively. Each merged cluster was then further dissected into 15 total subsets through subclustering based on median expression of both lineage and selected functional markers (**Table 1**).

**Table 1:** Immune cell lineage and functional markers used for cell profiling.

Lineage	Lineage markers	Functional/differentiation markers
B cells	CD19, CD20, IgD, CD27, CD38, CD21	CD40, HLA-DR, IL7Ra, CXCR5, CD79B, CD86, PD-1, CD69, CD11c, CD107, CD25, CXCR3
CD4+ T cells	CD3, CD4, CD45RA, CD45RO, CCR7, CD27	CD28, CD69, CD25, CD40L, CCR7, PD-1, IL7Ra, CXCR3
CD8+ T cells	CD3, CD8, CD45RA, CD45RO, CCR7, CD27	CD69, CD28, CXCR3, PD-1, CD38, IL7Ra, CD40L, CD25
Myeloid	CD11b, CD16, CD14, CD123, CD11c, CD66b	CD69, CD107a, CD86, HLA-DR, CD40, CD38, TIM3, CD45RA, CXCR3, CXCR5, CD40L, CD25
NK cells	CD16, CD56, CD38	CD8, CD45RA
TCRgd cells	CD3, TCRgd	CD27, CD69, CD45RO, CD45RA, CD28, CXCR3, PD-1, CD8, CD16

Fresh tumor, adjacent lung tissue, and PBMC samples from treatment-naïve LUAD patients were profiled by CyTOF using a 44-marker panel. Expression of specific lineage markers were used to define major immune cell populations (B cells, CD4+ T cells, CD8+ T cells, NK cells, TCRγδ T cells, and myeloid cells), and functional/differentiation markers were applied to further resolve cell activation states and phenotypes.

### 2.6 Human IgG/IgM adaptive immune receptor repertoire sequencing (AIRR-seq)

RNA was extracted from matched PBMC, tumor, and normal tissue samples of 29 patients from the CyTOF cohort, using RNeasy Mini Kit (Qiagen) according to manufacturer instructions. Yields were measured using Qubit RNA High Sensitivity Kit (ThermoFisher Scientific) and integrity was measured using the Bioanalyzer RNA Nano Kit (Agilent Technologies) and stored at -80C. RNA was prepared for sequencing using the SMARTer Human BCR IgG IgM H/K/L Profiling Kit (Takara Bio USA) according to manufacturer instructions with no modifications. Briefly, approximately 100ng of RNA was used for PBMC samples and 400ng for tumor and adjacent tissues. IgG and IgM primers were used to amplify heavy chain transcripts through a 5’RACE reaction. Library quality was measured using the 2100 Bioanalyzer High Sensitivity DNA Assay Kit (Agilent Technologies) and the Qubit 3.0 Fluorometer dsDNA High Sensitivity Assay Kit. Sequencing was done using the 600-cycle MiSeq Reagent Kit v3 (Illumina) on the MiSeq platform using 300bp paired-end reads. Samples were equimolar pooled into multiplexes of 24, ensuring all tissues and isotypes from each patient were grouped together, when possible. FASTQ reads were generated using the associated DRAGEN software package (Illumina).

### 2.7 Full length AIRR-seq human BCR IgG sequencing

Targeted amplification of IgG heavy chain transcripts was done using a novel near-full length sequencing method as described previously (28). Briefly, extracted RNA from a subset of patient tumor samples (n=21) used in CyTOF and traditional AIRR-seq was thawed on ice. Approximately 100–600 ng of RNA was used per sample and converted to first strand complementary DNA (cDNA) using the SMARTer RACE 5’/3’ Kit (Takara Bio USA) according to manufacturer instructions. A custom template switch oligonucleotide containing a unique molecular identifier (5’ TSO-UMI) was used for template switch during cDNA synthesis. PCR amplification of heavy chain transcripts was done using 10uL 5X PrimeSTAR GXL buffer, 4uL GXL dNTP mixture, 28uL PCR-grade water, 1uL PrimeSTAR GXL Polymerase, 1uL 10X UPM from the SMARTer RACE 5’/3’ kit and 1uL of appropriate barcoded IgG-specific 3’ primers (**Supplementary Table 2**). Amplification conditions for full-length IgG were done as previously described (26). Amplified products were purified using a 1x1 cleanup with ProNex magnetic beads (Promega) and quantified using Qubit dsDNA HS assay (ThermoFisher Scientific) and length was evaluated using the Fragment Analyzer Genomic DNA HS assay (Agilent). Amplifications were equimolar pooled into eight-plexes and prepared for sequencing using SMRTbell Express Template Prep Kit 2.0 (Pacific Biosciences) according to the “Procedure and Checklist for Iso-Seq Express Template for Sequel and Sequel IIe systems” protocol starting at “DNA Damage Repair” with modifications as previously described. Final libraries were quantified using the Qubit dsDNA HS assay (ThermoFisher Scientific) and quality was evaluated using the Fragment Analyzer Genomic DNA HS assay (Agilent). Sequencing of each sample pool was performed on one SMRTcell 8M using primer v4 and polymerase v2.1 on the Sequel IIe system with 30hr movies. Reads were demultiplexed and high-fidelity circular consensus sequences (“HiFi reads”) were generated on the instrument for downstream analysis.

### 2.8 BCR repertoire sequencing analysis

Both short-read AIRR-seq and FLAIRR-seq datasets were processed and analyzed using tools and packages from the Immcantation Portal (29). Quality control processing of AIRR-seq datasets was done using pRESTO (30) functions, “FilterSeq trimqual” to remove bases with <Q20 read quality and “FilterSeq length” to remove reads shorter than 125 bp. Primer sequences were identified with an error rate of 0.2 and noted in FASTQ headers using “MaskPrimers align.” Reads with the same 12 bp UMI were aligned using “AlignSets muscle” and a consensus sequence for each UMI was generated using “BuildConsensus.” Mate pairing was done with “AssemblePairs sequential” using reference genome-guided alignment with a minimum 5bp overlap and reads <400bp were removed using “FilterSeq.” Reads with the same UMI were collapsed using “CollapseSeq” and reads with <2 supporting sequences were removed using “SplitSeq group.” For pRESTO processing of FLAIRR-seq datasets, HiFi reads with <Q20 read quality and/or reads with <750 bp were removed using “FilterSeq quality” and “FilterSeq length.” Primers and a 22bp UMI were identified using “MaskPrimers align” with an error rate of 0.3 and reads with the same UMI were grouped and aligned using “AlignSets muscle.” FLAIRR-seq reads are done as a single molecule so mate pairing was not required. Subsequent quality control and filtration of consensus reads was done as described above. Filtered reads for both datasets were further processed using the Change-O (29) toolkit. For gene assignment, we used "IgBLAST" to align sequences against immunoblobulin heavy chain V (IGHV), D (IGHD), and J (IGHJ) alleles downloaded from the International IMmunoGeneTics Information System (IMGT) database (downloaded Febuary 21, 2022). Clonal clustering was performed with “DefineClones.py” followed by germline reconstruction and conversion using “CreateGermlines.py.” To define immunoglobulin heavy chain constant (IGHC) genes from FLAIRR-seq data, reads were filtered by IGHC length (900-1100bp) and aligned to chromsome 14 hg38 reference using minimap2 (31) and SAMtools (32) to generate sorted and indexed bam files. WhatsHap (33) was used to identify, genotype, and phase single nucleotide variants (SNVs), then reads from each gene were clustered using CD-HIT (34) with a 100% identity threshold. A single representative read from clusters representing at least 5% of the total reads per IGHC gene were aligned to an in-house IGHC reference dataset using BLAST (35) to confirm the IGHC gene assignments and infer alleles.

All repertoire-derived metrics were analyzed in R (v4.3.1) using functions from Alakazam (29), dplyr, and stats. Simpson’s diversity index was computed using “alphaDiversity” and 95% confidence intervals were obtained using 200 bootstrap replicates implemented within the function. Repertoire polarization was calculated using “countClones” to identify the minimum number of clones representing 80% of sequences, normalized to the total clone count. SHM frequencies were computed with "observedMutations" to calculate the number of abse changes between the full sequence alignment or specified region (complementarity determining regions and framework regions) and the constructed germline alignments.

IGHV, IGHD, and IGHJ gene usage frequencies were calculated using “countGenes.” To assess tumor enrichment, the frequency of each gene in PBMC IgM was subtracted from its frequency in tumor IgM or tumor IgG for each individual. Clone sizes for enriched versus depleted genes were compared by normalizing clone counts to total productive sequences per isotype and tissue. Principal component analysis (PCA) of gene-usage frequencies was conducted with “prcomp.” Differences in repertoire structure were quantified by calculating Euclidean distances along PC1–PC2 between (1): each sample and its nearest neighbor of the same tissue type, and (2) paired PBMC–tumor samples from the same patient.

Statistical comparisons between paired tissues were performed using paired t-tests, and differences between patient groups were compared using unpaired t-tests. When multiple genes, groups, or subisotypes were tested simultaneously, p-values were adjusted using Bonferroni correction to control for multiple hypothesis testing and significance thresholds after correction are reported in the text or figure legends.

### 2.9 Study approval

All human samples were obtained from LUAD patients under protocols approved by the University of Louisville School of Medicine Institutional Review Board, in accordance with the Declaration of Helsinki. Written informed consent was obtained from all participants prior to sample collection. For all patient data and images, consent was obtained for use in research, and records of consent have been retained. No identifying facial material is included.

## 3 Results

### 3.1 Increased density of CD19+ B cells in tumor tissues from LUAD patients

We analyzed tumor and matched non-tumoral adjacent lung tissue from a pilot cohort of 12 patients with LUAD (**Supplementary Table 3**). Flow cytometry revealed a significantly higher frequency of CD19+ B cells within tumors compared to adjacent tissue (**Supplementary Figures 1A, B**, paired t-test; *p*=8.02x10^-5^). Within the B-cell compartment, tumors were enriched for CD19+CD27+CD38+ (*p*=1.72x10^-5^) and CD19+CD69+ (*p*=0.033) subsets, whereas CD19+CXCR5+ (*p*=0.002) B cells were reduced relative to adjacent tissue (**Supplementary Figure 1C**; paired t-tests). Additionally, confocal microscopy of a subset of these patients revealed increased co-localization of CD3+ and CD19+ cells in tumor compared to adjacent tissue (**Supplementary Figure 1D**), consistent with previous studies (8, 11, 19, 36). Together, these initial findings highlighted an altered B cell population in LUAD tumors and underscored the need to investigate at higher resolution how B cell subsets and their expressed antibodies may shape the tumor microenvironment.

### 3.2 Peripheral blood, tumor, and non-tumoral adjacent lung tissue from LUAD patients have distinct immune cell profiles

**Figure 1:**
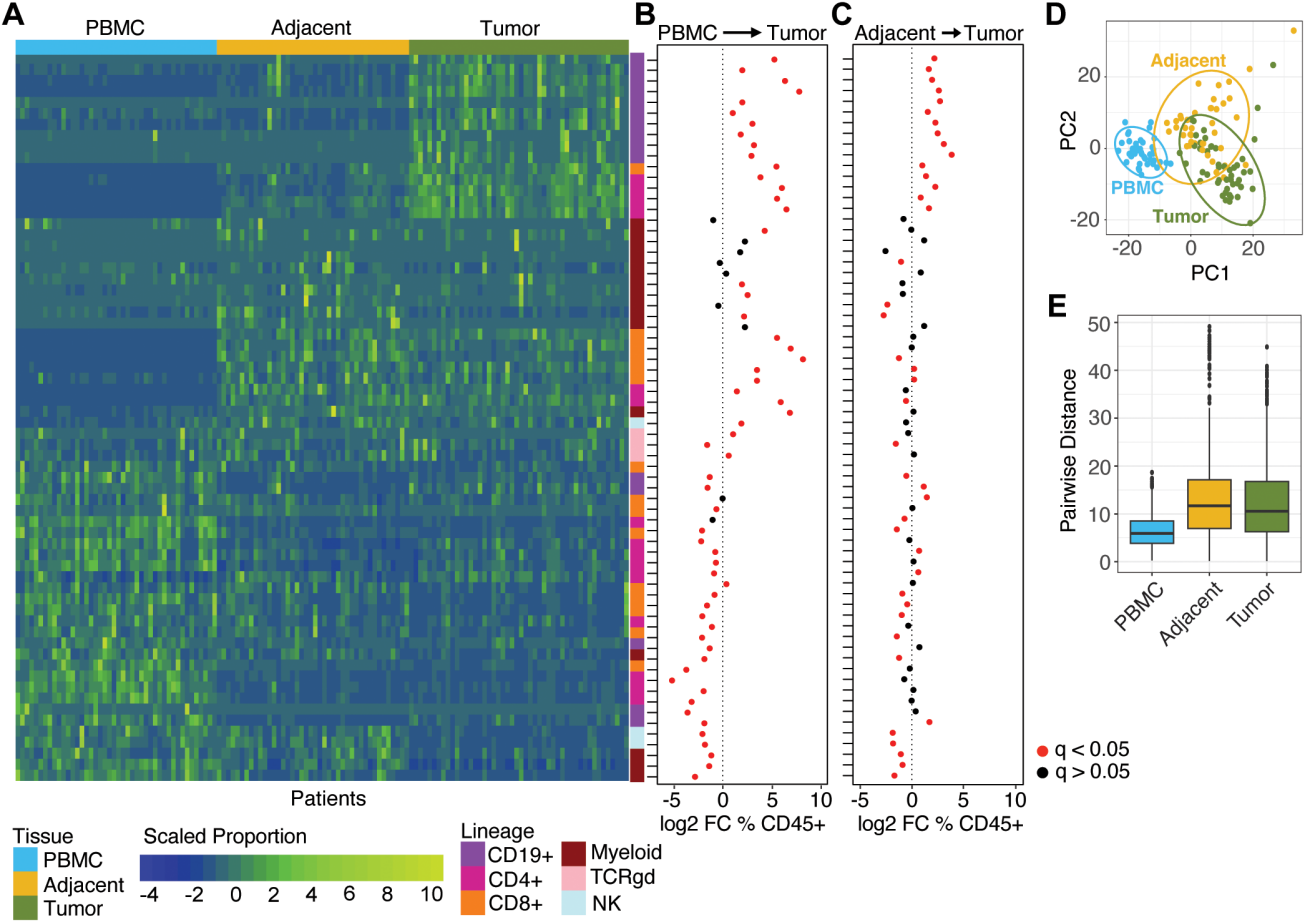
CyTOF-based immune cell subset distributions across matched tissues of LUAD patients. **(A)** Heatmap showing the relative proportions of 66 immune cell subsets across PBMC (n=44), adjacent (n=42), and tumor (n=48) tissues. Blue indicates lower proportions; yellow indicates higher proportions. **(B)** Log2 fold-change of immune cell subset proportions between matched PBMC and tumor samples (n=44). Positive values indicate enrichment in tumor; negative values indicate enrichment in PBMC. Significance was determined by paired t-tests with Bonferroni correction; red points denote statistically significant changes (q < 0.05). **(C)** Log2 fold-change of immune cell subset proportions between matched adjacent and tumor samples (n=42). Positive values indicate enrichment in tumor; negative values indicate enrichment in adjacent. Significance was determined by paired t-tests with Bonferroni correction; red points denote statistically significant changes (q < 0.05). **(D)** Principal component analysis (PCA) of immune cell subset proportions across PBMC, adjacent, and tumor samples. **(E)** Pairwise Euclidean distances between samples within each tissue type calculated from PC1 and PC2 coordinates.

We collected peripheral blood, tumor tissue, and non-tumoral adjacent lung tissue (adjacent) from 48 LUAD patients (I n=28; II n=12; III n=7; IV n=1), divided equally between male and female (**Table 2**). All patients were treatment naive or had not received treatment at least 65 days prior to surgery. To characterize cell populations, we performed CyTOF analysis using a panel of 44 immune cell surface markers (**Supplementary Table 1**), allowing for resolution of major T cell, B cell, NK, and myeloid-derived subpopulations. On average, we processed 2.12x10^5^ live cells per tissue sample (range = 3.73x10^4^ – 4.54x10^5^), with approximately 40% of events recorded identified as CD45+ (mean = 8.2x10^4^, range = 5.17x10^3^ – 2.43x10^5^). Using defined lists of immune cell lineage markers (**Table 1**), we first identified a total of 20 cell clusters composed of both myeloid and lymphoid lineages, in which we identified two CD3+CD8+ clusters, two CD3+CD4+ clusters, two CD3-CD19+ clusters, three CD16+CD56+CD3-CD19- clusters, three CD3+TCRgd+ T cell clusters, and six CD11b+ clusters (**Supplementary Figure 2**). Clusters with expression patterns associated with each primary lineage (CD4+ T cells, CD8+ T cells, CD11b+ myeloid cells, CD19+ B cells) were merged and re-clustered using markers specific to that cell type (**Table 1**; **Supplementary Figures 3–6**). Overall, this resulted in the identification of 66 distinct immune cell clusters across patients and tissues (**Figure 1A**).

**Table 2:** Clinical characteristics of LUAD patient cohort in CyTOF analysis.

Characteristic	# of patients (n=48)
Gender	
Female	24
Male	24
Stage	
I	28
II	12
III	7
IV	1
Age	
mean	65.5
range	35-84

Fresh tumor, adjacent, and PBMC samples were obtained from treatment-naïve early-stage LUAD patients (n = 48) undergoing surgical resection at the James Graham Brown Cancer Center, University of Louisville. Patients were either treatment-naïve or 6 months out from treatment. Samples were collected under Institutional Review Board approval with written informed consent.

We next sought to identify cell subsets that were enriched in patient tumors. To do this, we tested for paired differences in cell proportions between tissues within patients. We identified 57 cell subsets with different proportions between PBMC and tumor tissues (paired t-test with Bonferroni correction, *q*<0.05), 31 of which were specifically elevated in tumor relative to PBMC (**Figure 1B**). Of these, 14 were not different when comparing tumor to adjacent tissue indicating that their increased proportions relative to PBMC likely reflected recruitment to lung tissue generally and not to lung tumors specifically. We identified 39 subsets with significantly different proportions between matched tumor and adjacent tissue (paired t-test; Bonferroni, *q*<0.05), the majority of which (n=22, 53.8%) were elevated in tumor (**Figure 1C**). Notably, of those 21 subsets, 16 (76.2%) were among those found to also be elevated in tumor relative to PBMC. Additionally, 12 subsets were found to be significantly depleted in tumor on average, relative to both adjacent tissue and PBMC (**Figures 1B, C**). These cells included senescent CD8+ cells (CD45RO-CD45RA-CCR7-CD27-), as well as 2 NK subsets and 4 myeloid cells (classical monocytes, dysfunctional pDCs, cDCs, and granulocyte precursor cells).

We next focused on the 16 subsets whose proportions were elevated in tumor relative to both tumor-adjacent tissue and to PBMC, a pattern consistent with tumor infiltration. Notably, a majority, 10 (62.5%), of these tumor-infiltrating subsets were B lymphocytes corresponding to a variety of differentiated subtypes. These included resident activated memory B cells (CD19+CD20+IgD-CD21+CD27+CD69+), IgD+ activated memory B cells (CD19+CD20+IgD+CD21+CD27+CD69-), antigen presenting B cells (CD19+CD20+IgD-CD27-CD38+CD21+CD86+PD-1+), activated B cells (CD19+CD20+IgD-CD21+CD69-CD11c+CD86loCD79Blo), activated memory B cells (CD19+CD20+IgD-CD27+CD38-CD21lo), atypical resident B cells (CD19+CD20+IgD-CD27-CD38-CD21loCD69+), resident memory (CD19+CD20+IgD-CD27+CD38-CD21-CD69+), plasma cells (CD19+CD20-IgD-CD27+CD38++CD21-), IgD+ plasma cells (CD19+CD20-IgD+CD27+CD38++CD21-), and terminally differentiated plasma cells (CD19loCD20-IgD-CD27+CD21-CD38++). The remaining 6 subsets included CD4+CD69+ effector memory T cells (CD45RO+CCR7-CD27+CD69+), CD4+ CD69+ central memory (CD45RO+CCR7+CD27+CD69+), two regulatory CD4+ T cell subsets (CD45RO+CCR7-CD27+CD69+CD25+), CD8+ resident memory T cells (CD45RO+CCR7-CD27+CD69+) and CD8+ CD69+ TEMRA cells (CD45RA+CD45RO-CCR7-CD27+).

PCA using the proportions of the 66 subsets revealed distinct profiles for each tissue (**Figure 1D**). Based on principal components (PCs) 1 and 2 there was partial overlap between tumor and normal adjacent tissue clusters; however, PBMC samples formed a distinct cluster along PC1, which explained 34.7% of variation across samples. In contrast, adjacent and tumor tissues separated along PC2, which only explained 14.1% of variation across samples. PCA also revealed that the average inter-patient distances when comparing PBMC were much lower than those for either tumor or tumor-adjacent tissues (**Figure 1E**). Variability within tumor samples was especially pronounced for the 16 cell subsets whose enrichment patterns indicated they were tumor-infiltrating (**Supplementary Figure 7**). Hence, background interindividual variability seems likely to explain only a small part of the variability found in TMEs.

### 3.3 Distinct patient groups are defined by variation in tumor-infiltrating immune profiles

Our observation that tumor immune profiles are highly variable between patients (**Figures 1D, E**; **Supplementary Figure 7**) is consistent with previous studies that have shown variation in the numbers of tumor-infiltrating and tertiary lymphoid structure-associated immune cells among LUAD patients (8, 11, 19, 36, 37). Notably, many previous studies have noted the prognostic value of B cells in LUAD (12, 19, 38), and their potential interactions with other immune cell types, including CD4+ and CD8+ subsets (8, 11, 36). However, for the majority of these studies, the lower resolution profiling of immune sub-populations, particularly B cells subsets, has led to demarcation of a limited number of patient groupings with inconsistent prognostic value.

**Figure 2:**
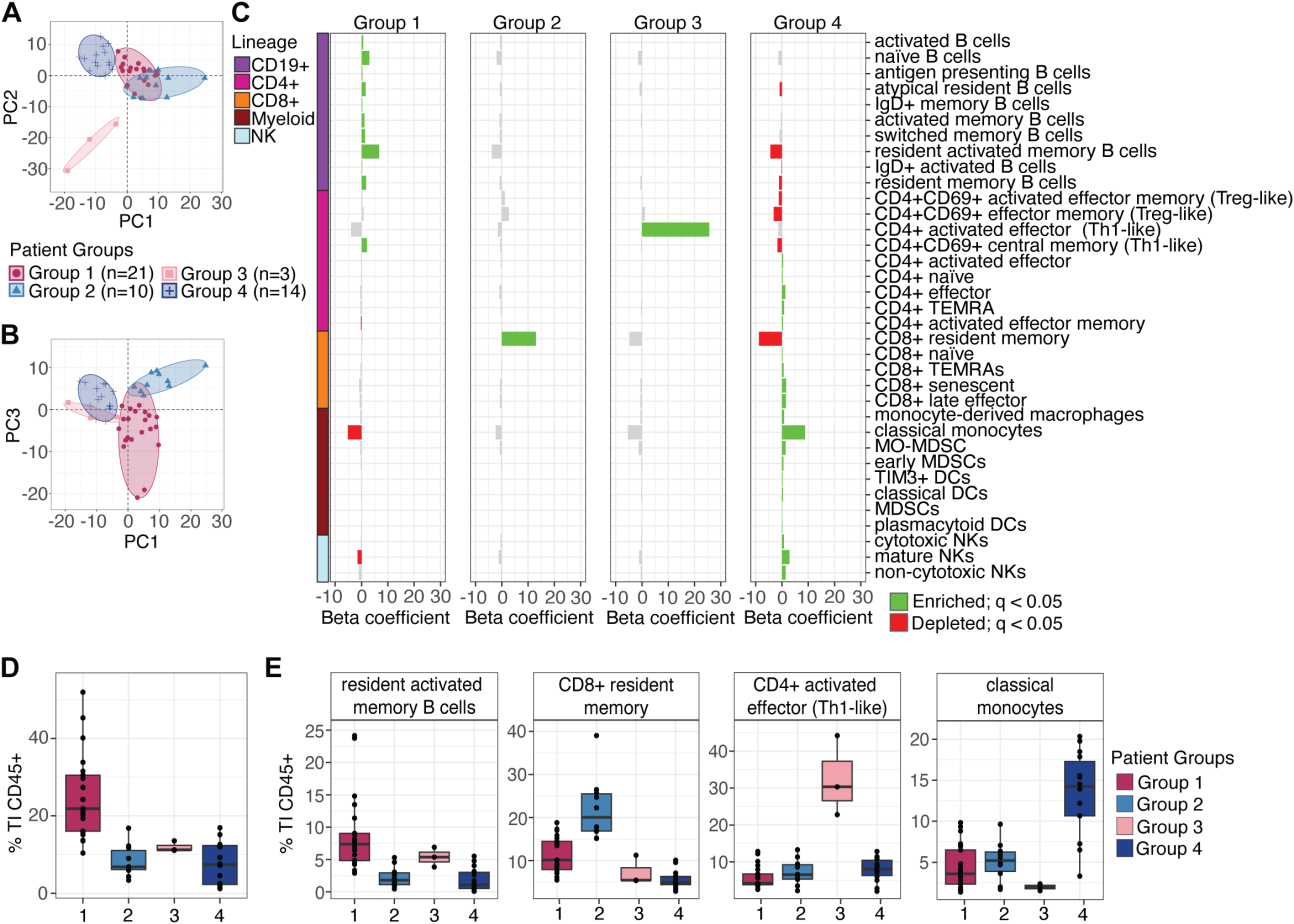
Unsupervised clustering of patients based on tumor-infiltrating immune cell subset proportions. **(A)** Principal component analysis (PCA) showing components 1 and 2 for patient groups defined by k-means clustering of tumor-infiltrating (TI) immune cell proportions. Ellipses encompass all patients in each identified group (Group 1, n=21; Group 2, n=10; Group 3, n=3; Group 4, n=14). **(B)** PCA plot of components 1 and 3, with ellipses as in **(A)**. **(C)** Relative proportions of 35 immune cell subsets that were significantly increased or decreased in one patient group compared to all others, determined by linear regression. Green bars, subsets enriched in the indicated group; red bars, subsets decreased. **(D)** Proportion of total B cells (CD19^+^) in tumor samples across the four patient groups. **(E)** Relative proportions of dominant tumor-infiltrating T cell and myeloid subsets by group: resident activated memory B cells in Group 1, CD8+ resident memory in Group 2, CD4^+^activated effector (Th1-like) in Group 3, and classical monocytes in Group 4.

Here we sought to identify consistent tumor-infiltrating immune cell profiles among groups of patients, by performing unsupervised k-means clustering of our patients using tumor tissue proportions of the 66 CD45+ immune cell subsets described above. Using this approach, we identified 4 groups of patients (Groups 1-4), each characterized by distinct TI immune cell profiles. Using PCA, variation among patients and their respective assigned clusters was largely explained by PC1 (26.99%), PC2 (20.44%), and PC3 (15.34%; **Figures 2A, B**). We next used linear regression to determine which cell subsets explained the most variation in a given patient group relative to all other groups combined; this allowed us to effectively identify cell subsets that were either significantly enriched or depleted in each group. In total, we identified 35 immune cell subsets that wre significantly enriched or depleted (Bonferroni, *q*<0.05) in at least one group (**Figure 2C**; **Supplementary Figure 8**). In addition, to ensure these cell subsets were indicative of patient subgroup rather than other clinical features of these patients, we confirmed the lack of association with patient stage, biological sex, and age (**Supplementary Figure 9**; **Table 3**).

**Table 3:** Association of immune cell profiles with clinical features.

Clinical feature	x^2^	df	*P*-value
Smoking status	7.9467	6	0.242
Stage	7.1701	9	0.6194
Gender	2.3048	3	0.5116
Age	15.983	15	0.3832

Chi-squared tests were used to evaluate associations between patient TI immune cell group and clinical features, including smoking status, stage, gender, and age. No significant associations were observed (p > 0.05 for all comparisons).

Patients in Group 1 had significantly higher proportions of 10 B cell subsets, including resident activated memory B cells (beta coeff. = 6.66; *q*=4.13^-5^), naïve B cells (beta coeff. = 1.09; *q*=0.005), and antigen presenting B cells (beta coeff. = 0.272; *q*=0.013). Notably, 7 of these B cell subsets were among the 16 originally found to be enriched in tumors when we collectively analyzed all patients in this cohort (**Figure 1B**). The increased proportion of these B cell subsets in Group 1 was also reflected by the increased proportions of total B cells (CD19+ cells) relative to all other groups; indeed, in some Group 1 patients, CD19+ proportions represented >40% of all tumor-infiltrating CD45+ cells (**Figure 2D**). In addition, these increased B cell subsets were also associated with significantly higher proportions of CD4+ CD69+ central memory T cells (beta coeff. = 2.04; *q*=0.010) in Group 1 (**Figure 2C**). This combination of TI immune cell subsets, particularly the elevated representation of B cell populations was consistent with previous reports (8, 11). It is these particular B cell subsets that, when found enriched in tertiary lymphoid structures, have been associated with improved patient outcomes (8, 11, 19, 36). In contrast to Group 1 enriched cell subsets, CD4+ activated effector memory (beta coeff.=-0.270; *q*=0.024), classical monocytes (beta coeff.=-5.23; *q*=0.005), and mature NK cells (beta coeff.=-1.57; *q*=0.043) were moderately lower in Group 1 relative to the other groups combined; however, the proportions of these subsets in Group 1 were not uniquely lower when compared individually to Groups 2 and 3 (**Supplementary Figure 10**).

For Group 2 patients, tumors were significantly enriched for CD8+ resident memory T cells (beta coeff. = 13.0; *q*=4.22^-7^), whereas the tumors of Group 3 patients were found to be uniquely enriched for CD4+ activated effector (Th1-like; beta coeff. = 25.6; *q*=9.95^-13^) (**Figure 2C**). In both instances, these cell subsets were dominant subsets among tumors of Groups 2 and 3, respectively (**Figure 2E**). For example, in all three individuals of Group 3, CD4+ activated effector (Th1-like) cells represented >20% of all CD45+ subsets, compared to the other three patient groups in which the mean was <10% (**Figure 2E**). Finally, tumor profiles of Group 4 patients were dominated by myeloid cell populations, including classical monocytes (beta coeff. = 8.79; *q*=4.73^-9^) and early myeloid-derived suppressor cells (early MDSCs) (beta coeff. = 1.36; *q*=0.018), as well as multiple NK cell populations, including mature NK cells (beta coeff. = 2.86; *q*=1.19^-5^). In addition, many T lymphocyte populations were elevated in Group 4 relative to groups 1, 2, and 3; however, among these cell types there was biased representation of naïve and TEMRA subsets. These included naive CD4+ (beta coeff. = 0.294; *q*=0.035) and CD8+ (beta coeff. = 0.224; *q*=0.019) T cells, and senescent CD8+ T cells (beta coeff. = 1.61; *q*=0.003). Collectively, this signature was suggestive of an immunologically inactive tumor, consistent with earlier studies, potentially associated with the formation of myeloid-derived suppressor cells (43).

### 3.4 Variation in the proportions of B cell subsets further differentiate patient groups

The most notable signature differentiating Group 1 from Groups 2–4 was the overall enrichment of CD19+ B cells among CD45+ cells (**Figure 2D**). This observation was consistent with previous reports showing elevated CD19/20+ B cells in some LUAD patients (8, 11, 19, 36). However, given the increased subset resolution possible with our dataset, we sought to further define the B cell populations underlying this enrichment. Specifically, we asked whether in addition to the enrichment of B cells in the tumor overall among Group 1 patients, whether the distribution of subsets within CD19+ cells was also distinct in Group 1. We reasoned that within-B cell subpopulation differences might explain why Group 2 and Group 3 show higher representation of CD8+ and CD4+ T cell subsets, further supporting differential immune cell interactions across groups.

**Figure 3:**
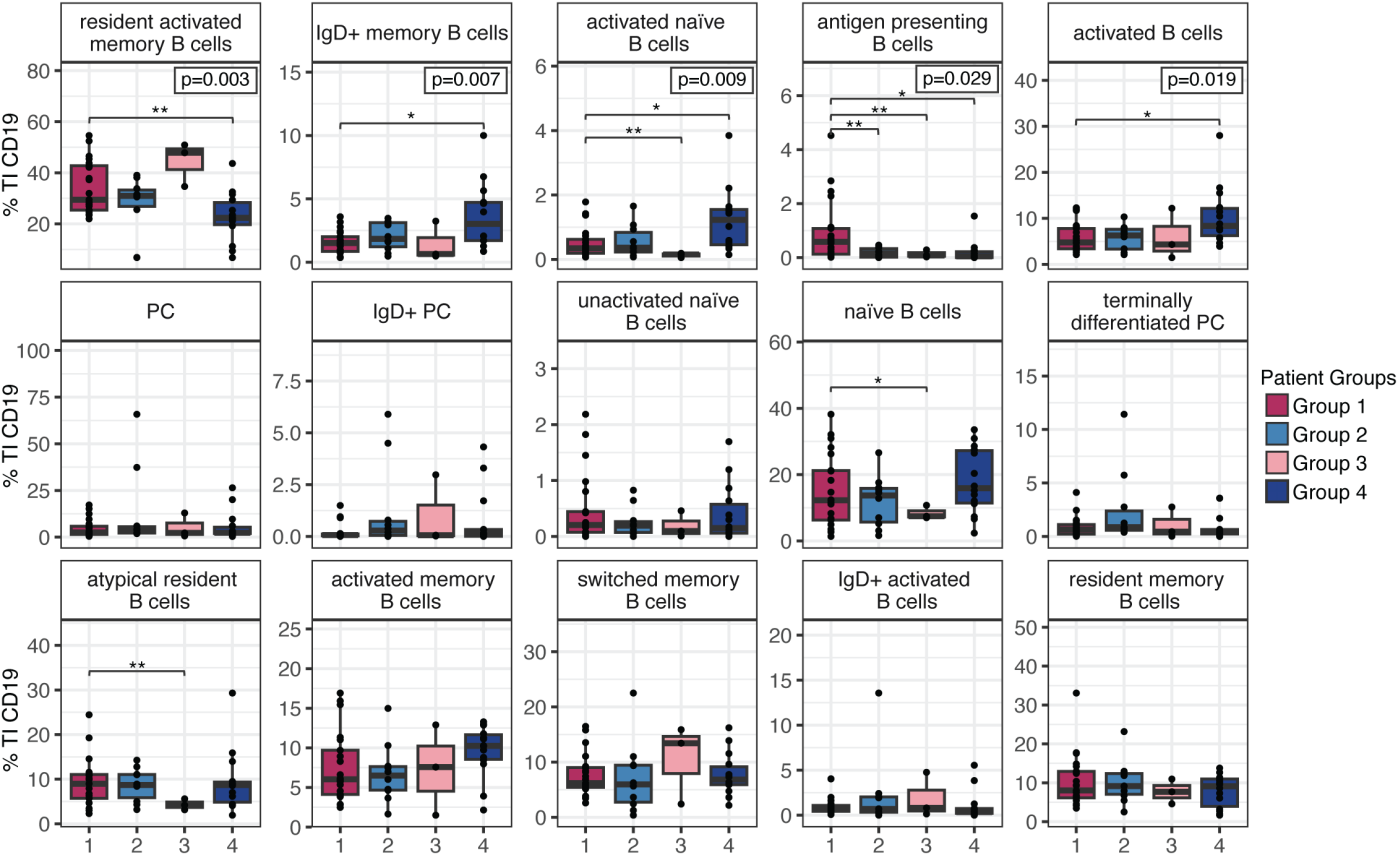
B cell subset proportions normalized to total tumor-infiltrating B cells across patient groups. Proportions of 15 CD19^+^ B cell subsets within the tumor-infiltrating (TI) B cell compartment were compared across LUAD patient groups defined by TI immune profiles (Groups 1–4; n=48). Five subsets showed significant variation between groups — activated memory B cells, IgD^+^ memory B cells, inflammatory B cells, regulatory B cells (Bregs), and antigen-presenting B cells with significance determined by ANOVA (*p* < 0.05). Pairwise differences between groups were assessed by unpaired t-tests and significance is indicated as follows: *p < 0.05; **p < 0.01.

Of the 15 B cell clusters identified, we identified 5 that showed statistically significant variation between the four patient groups. These included, resident activated memory B cells (ANOVA, *p* = 0.003), IgD+ memory B cells (ANOVA, *p* = 0.007), activated naive B cells (ANOVA, *p* = 0.009), activated B cells (ANOVA, *p* = 0.019), and antigen presenting B cells (ANOVA, *p* = 0.03) (**Figure 3**). Group 1 and Group 2 patients exhibit similar proportions across most B cell subsets, with a significant difference observed only in antigen presenting B cells (student’s t-test *p*<0.01; **Figure 3**). In contrast, Group 3 patients show notably lower proportions in four B cell subsets (antigen presenting, activated naive, atypical resident, and naive B cells) compared to Group 1 (student’s t-test *p*<0.05; **Figure 3**). Group 4 stands out as the most divergent, with two subsets (resident activated memory and antigen presenting B cells) present at lower proportions, and three subsets (IgD+ memory, activated naive, and activated) at significantly higher proportions relative to Group 1 (unpaired t-test *p*<0.05; **Figure 3**). Together these results demonstrate that not only are B cells elevated in particular patient groups, but that within the B cell compartment, there are specific distributions of B cell subpopulations.

### 3.5 The tumor BCR repertoire is characterized by increased clonal expansion, somatic hypermutation, and biased IGH gene usage

**Figure 4:**
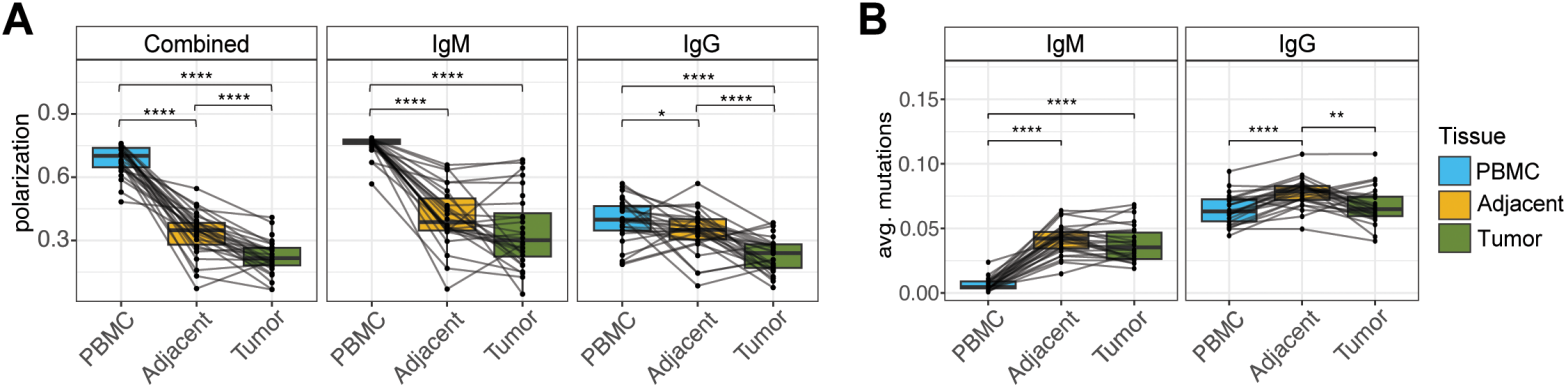
Evidence of clonal expansion in tumor IgM and IgG revealed by AIRR-seq analysis. **(A)** Repertoire polarization compared across tissues using paired *t* tests with all sequences (left; n=26), IgM sequences alone (center), and IgG sequences alone (right). **(B)** Somatic hypermutation (SHM) frequency compared across tissues for IgM (left; n=26) and IgG (right; n=26) using paired *t* tests. Significance is indicated as follows: *q < 0.05; **q < 0.01; ****q < 0.0001.

To extend our investigation of B cell mediated immune responses in the LUAD TME, we next sought to characterize tumor-associated signatures in the expressed BCR repertoire. We conducted bulk AIRR-seq on IgM and IgG repertoires of matched PBMC, adjacent tissue, and tumor tissue from a subset of patients from the cohort studied above (n=29; **Supplementary Table 3**); this included 12, 7, and 4 individuals from Groups 1, 2, and 4 respectively. Given the infiltration of B cells noted in tumors (**Figure 1**), we reasoned that there would be associated changes in the BCR repertoire providing evidence of B cell activation and an antigen-driven immune response. We assessed this by comparing measures of clonal expansion and SHM between tissues. Using the repertoire polarization metric (39), defined as the number of clones that represent 80% of the total sequenced repertoire, we found that compared to PBMC, both adjacent tissue and tumor BCR repertoires were significantly more polarized (**Figure 4A**). This was true for both the combined IgM/IgG repertoire, as well as for IgM and IgG repertoires when considered separately (**Figure 4A**). Supporting this observation, we noted that repertoire diversity (Simpson’s diversity index) was also lower in adjacent tissue and tumor relative to PBMC (**Supplementary Figure 11A**).

We next assessed SHM frequency in the IgM and IgG repertoires across tissues. As expected, for all tissues we observed higher frequencies of SHM in the IgG repertoire relative to IgM (**Supplementary Figure 11B**). However, within each isotype-specific repertoire, we noted additional differences in SHM frequencies between tissues. For IgM, SHM frequencies in PBMC were low (mean = 0.004), likely reflecting the fact that the IgM repertoire captured in the periphery was largely representative of antigen-naive B cells. In contrast, SHM in both the adjacent and tumor samples was significantly higher (**Figure 4B**). Inter-tissue differences in IgG SHM frequencies were more subtle. However, SHM frequencies were significantly higher in adjacent tissue, relative to both PBMC and tumor repertoires (**Figure 4B**). These trends were consistent when we considered silent and replacement mutations separately; however, we did note a slight increase in the percent of replacement mutations (relative to silent mutations) in IgM and IgG for both PBMC and tumor repertoires compared to adjacent lung tissue (**Supplementary Figures 11C, D**). Collectively, these results provide evidence that tumor infiltrating B cells have undergone clonal expansion and potentially affinity maturation, suggestive of antigen-associated immune responses.

**Figure 5:**
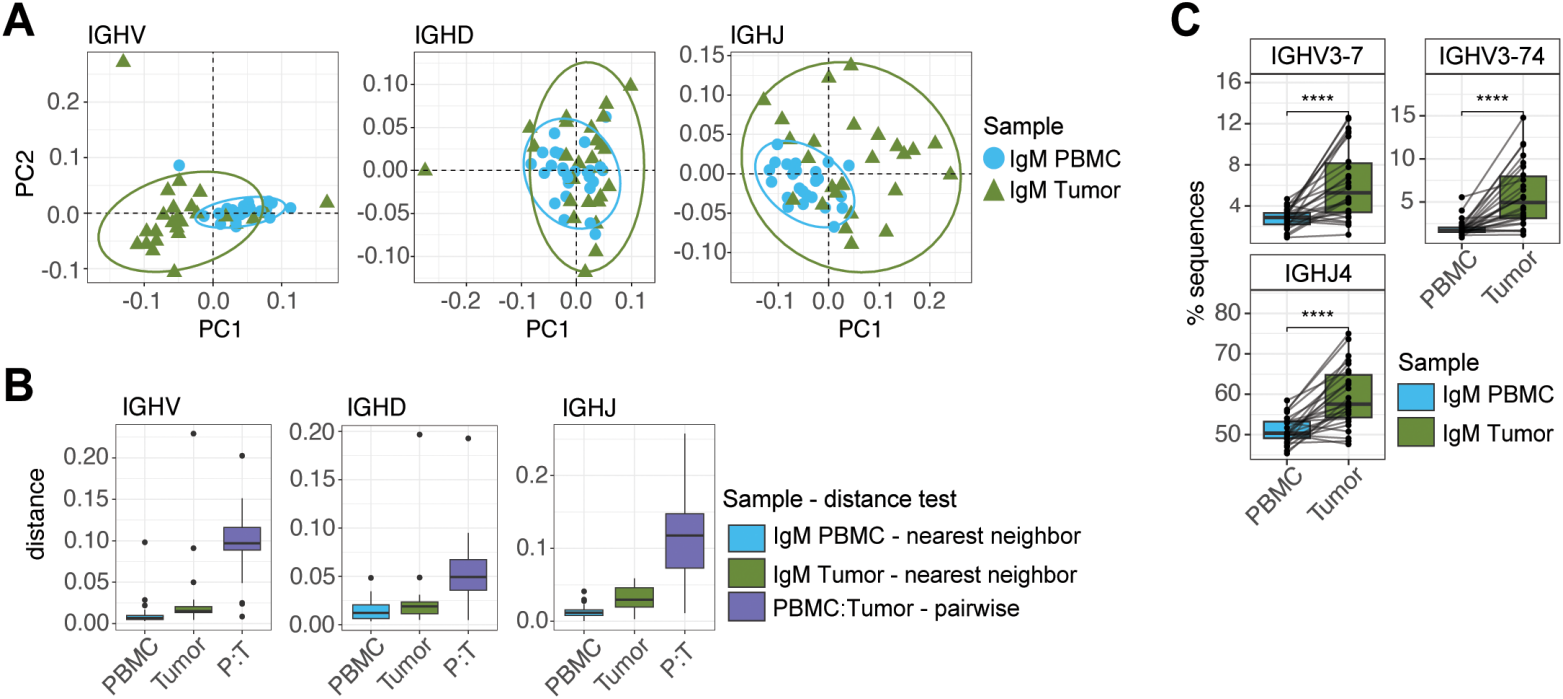
Distinct IGHV, IGHD, and IGHJ gene usage patterns in tumor IgM repertoires. **(A)** Principal component analysis (PCA) of IGHV (left), IGHD (center), and IGHJ (right) gene usage in matched PBMC and tumor IgM repertoires (n=26). **(B)** Nearest neighbor distances calculated between donors within tissues and pairwise distances between tissues within donors demonstrates patient-specific divergent repertoires. **(C)** IGHV and IGHJ genes significantly enriched in tumor IgM relative to matched PBMC IgM repertoires. Significance determined by paired t-tests with Bonferroni correction and indicated as follows: ****q < 0.0001.

To further examine differences between BCR repertoires in the tumor relative to those circulating in the periphery, we evaluated biases in heavy chain BCR gene usage in tumor IgM and IgG repertoires compared to PBMC IgM. We reasoned that genes enriched in tumor repertoires relative to those in the periphery could represent BCRs engaging tumor antigen. We calculated usage frequencies of IGHV, IGHD, and IGHJ genes in PBMC IgM, as well as tumor IgM and IgG separately. To first broadly compare IgM gene usage between tissues, we conducted PCA of IGHV, IGHJ and IGHD gene frequencies, in matched IgM PBMC and tumor samples (**Figure 5A**). This revealed that tumor and PBMC IgM repertoires had broadly differentiated gene usage profiles (**Figure 5A**). Specifically, nearest neighbor distances between repertoires within tissues were much lower than matched distances within donors between tissues (**Figure 5B**). We next sought to identify specific genes enriched in tumor IgM repertoires relative to PBMC IgM representing gene usage shifts common across donors. Based on a paired t-test, we identified 2 IGHV genes (IGHV3-7; IGHV3-74) and 1 IGHJ gene (IGHJ4) significantly enriched in tumor IgM after Bonferroni correction (**Figure 5C**). Additional IGHV, IGHD, and IGHJ genes were significantly depleted (**Supplementary Figure 12A**).

**Figure 6:**
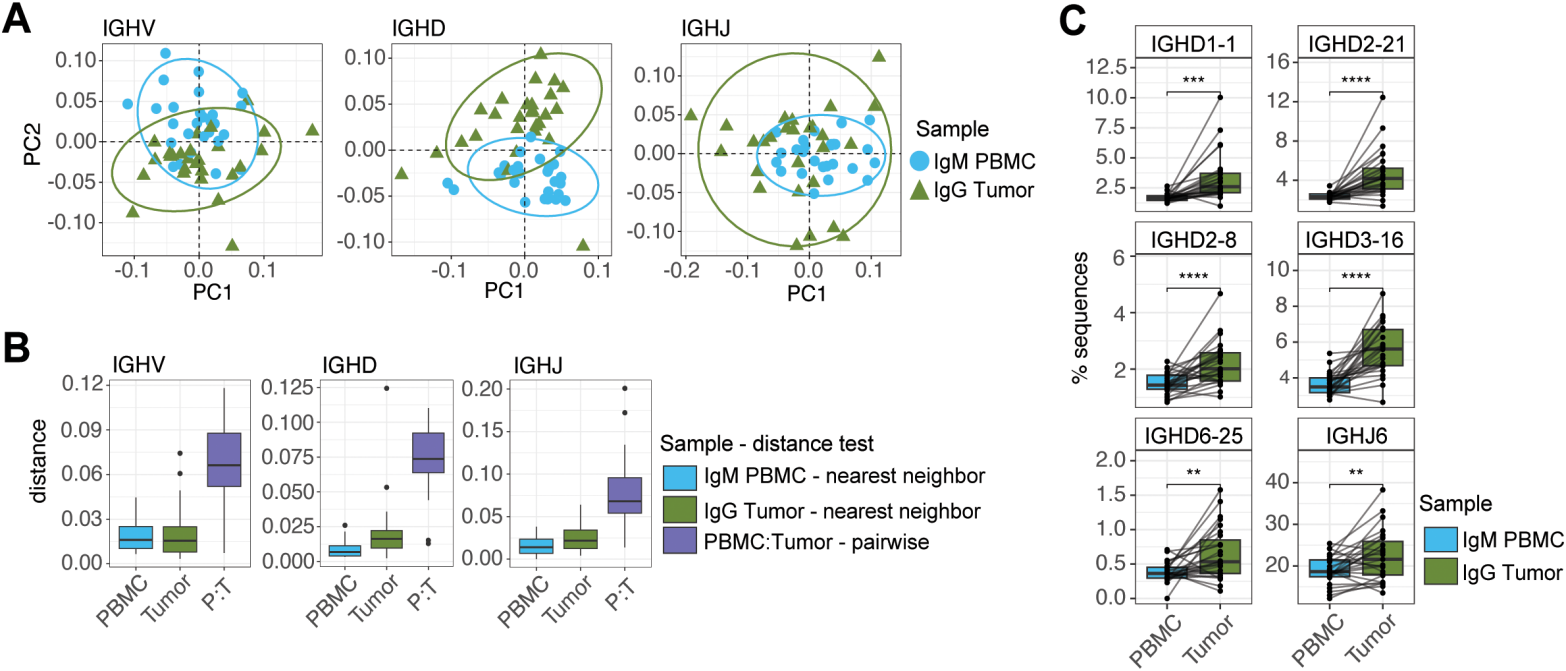
Distinct IGHV, IGHD, and IGHJ gene usage patterns in tumor IgG repertoires. **(A)** PCA and **(B)** pairwise distances of IGHV (left), IGHD (center), and IGHJ (right) gene usage in matched PBMC and tumor IgG repertoires (n=27). **(C)** Genes significantly enriched in tumor IgG compared with matched PBMC IgM repertoires (paired *t* test, Bonferroni-corrected *q* < 0.05; n=27). Significance is indicated as follows: **q < 0.01; ***q < 0.001; ****q < 0.0001.

We next compared PBMC IgM with tumor IgG repertoires, and PCA again demonstrated separation between the two tissues (**Figure 6A**). Nearest neighbor distances within tissues and pairwise distances within donors between tissues further supported that tumor IgG repertoires had diverged from PBMC IgM baselines (**Figure 6B**). Unlike tumor IgM, no IGHV genes were significantly enriched in tumor IgG relative to PBMC IgM, consistent with the notion that gene-usage shifts in tumor were not uniform across donors. However, five IGHD genes and one IGHJ gene exhibited significantly higher usage in tumor IgG, indicating some common patterns of change (**Figure 6C**). As with IgM, several IGHV, IGHD and IGHJ genes were significantly depleted in tumor IgG repertoires (**Supplementary Figure 12B**).

### 3.6 Tumor-enriched BCRs exhibit differences in V, D, and J gene usage, clone size, SHM, and CDR3 signatures

**Figure 7:**
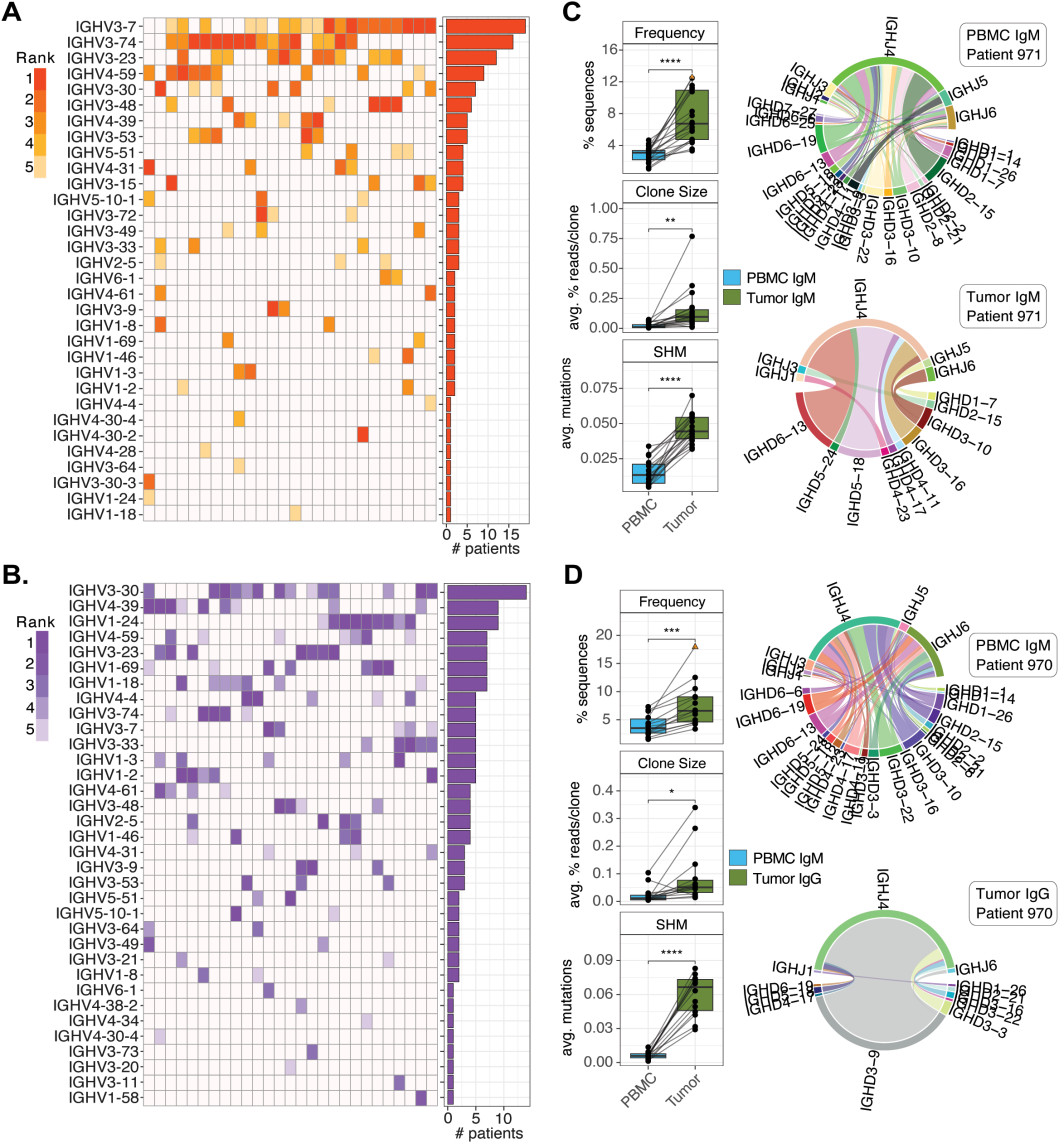
Patient-specific IGHV gene enrichment in tumor repertoires is associated with clonal expansion, SHM, and distinct CDR3 features. **(A, B)** Top IGHV genes enriched in tumor IgM (n=26) **(A)** and IgG (n=27) **(B)** versus PBMC IgM, shown per patient. Bars indicate the number of patients for which each IGHV gene ranked among the top five most enriched in tumor. **(C, D)** Representative IGHV genes enriched in tumor repertoires: IgM-enriched IGHV3-7 (n=19) **(C)** and IgG-enriched IGHV3-30 (n=14) **(D)**. Panels show relative IGHV usage in PBMC versus tumor (top left), clone size distribution (center left), SHM frequency (bottom left), and IGHD/IGHJ pairings in PBMC IgM (top right) and tumor (bottom right) from a representative patient. Statistical significance was determined by paired t-test and indicated as follows: *p < 0.05; **p < 0.01; ***p < 0.001; ****p < 0.0001.

While the genes highlighted in **Figures 5** and **6** represent those that exhibited average biases in tumor repertoires across donors collectively, this analysis did not account for genes that were commonly enriched in tumors of smaller subsets of patients. To assess this, we evaluated each patient individually, identifying the IGHV genes with the highest delta values between tumor IgM and IgG (separately) relative to PBMC IgM. In tumor IgM repertoires, 8 IGHV genes ranked among the top 5 most differentially used genes in at least 5 patients; the most frequently observed gene was IGHV3-7, identified in 19/26 patients (**Figure 7A**). Notably, this gene was also found to be significant overall in the matched comparison of PBMC and tumor IgM (**Figure 5C**). In tumor IgG, there was a greater number of IGHV genes (n=13) found among the highly ranked differentially used genes in 5 or more patients (**Figure 7B**), indicating again that the IgG tumor IGHV repertoire showed evidence of IGHV gene usage convergence only across smaller subsets of patients, rather than the majority of patients.

**Figure 8:**
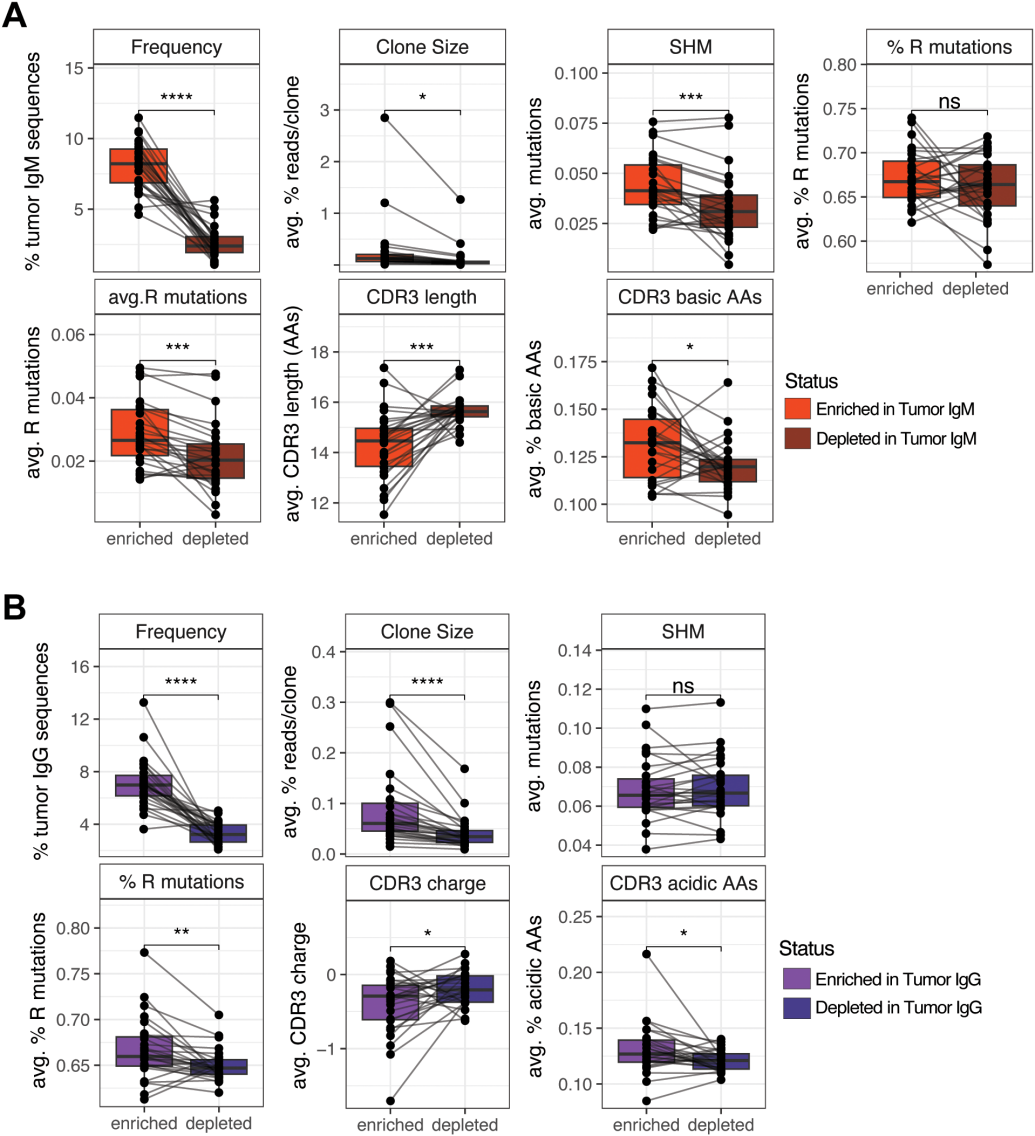
BCR features associated with tumor-enriched and depleted IGHV genes. Aggregate analysis of BCR features associated with enriched and depleted IGHV genes within individual patients for IgM (n=26) **(A)** and IgG (n=27) **(B)**. Statistical significance was determined by paired t-test and indicated as follows: ns, not significant (*p* > 0.05); *p < 0.05; **p < 0.01; ***p < 0.001; ****p < 0.0001.

To test whether tumor-enriched IGHV genes in each patient coincided with changes in additional signatures associated with antigen selection, we assessed differences in: 1) the size of clones represented by those genes compared between tumor IgM/IgG and PBMC IgM; 2) SHM patterns; and 3) D and J gene usage profiles. Examples provided for the top two most commonly enriched IGHV genes in both tumor IgM (**Figure 7C**; **Supplementary Figure 13A**) and IgG (**Figure 7D**; **Supplementary Figure 13B**) demonstrated that clones associated with these genes are larger, have higher SHM frequency, and at an individual level, exhibit biased D and J usage in tumor relative to PBMC. This indicated that genes enriched in tumor reflected recruitment of particular clones composed of specific rearrangements of V, D, and J gene segments that had specifically expanded and taken on SHM. Notably, these trends were observed when we collectively considered features of BCRs associated with all top enriched genes relative to their depleted counterparts within the same donors (**Figures 8A, B**). Similar to gene-level data shown in **Figures 7C, D**, we observed that tumor-enriched IgM BCRs were represented by larger clones and increased SHM (**Figure 8A**). The same was also noted for IgG BCRs with respect to clone size differences (**Figure 8B**). However, we noted differential SHM signatures were specific to increased replacement (‘R’) mutations (**Figure 8B**), rather than overall SHM. We also tested for additional differences in CDR3 characteristics, including length, overall charge, and the fraction of basic or acidic positions. For IgM, we specifically noted that CDR3 length was shorter on average in enriched BCRs, but that CDR3 sequences in these BCRs also had a greater proportion of basic amino acids (**Figure 8A**). Likewise, enriched IgG BCRs were also associated with differential CDR3 features, relative to depleted BCRs; however, these features were different from those observed for IgM (**Figure 8B**).

### 3.7 IgM and IgG BCR repertoires are more clonally expanded in patient groups with elevated levels of tumor-infiltrating B cells

**Figure 9:**
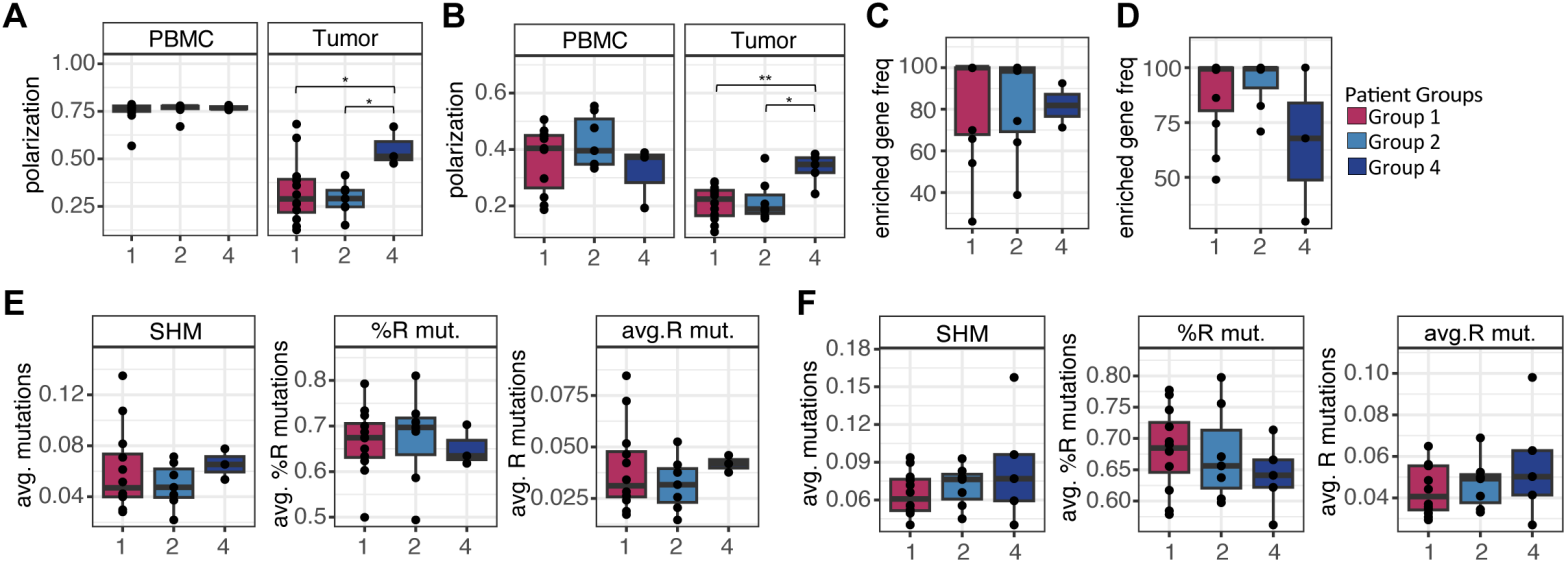
Clonal expansion and SHM patterns vary across patient groups. **(A)** Polarization of PBMC and tumor IgM repertoires compared across patient groups (unpaired t-test; Group 1, n=12; Group 2, n=7; Group 4, n=3). **(B)** Polarization of PBMC and tumor IgG repertoires compared across patient groups (unpaired t-test; Group 1, n=11, Group 2, n=7, Group 4, n=4). **(C)** Combined frequency of enriched genes per patient in the top 10% of expanded clones in tumor IgM compared across patient groups (Group 1 n=11, Group 2 n=7, Group 4 n=2). **(D)** Combined frequency of enriched genes per patient in the top 10% of expanded clones in tumor IgG compared across patient groups (Group 1 n=11, Group 2 n=7, Group 4 n=3). **(E)** Somatic hypermutation (SHM; left), percentage of replacement (‘R’) mutations (center), and frequency of R mutations (right) in the top 10% largest clones from tumor IgM. **(F)** Somatic hypermutation (SHM; left), percentage of replacement (‘R’) mutations (center), and frequency of R mutations (right) in the top 10% largest clones from tumor IgG. Significance was determined by paired t-tests and indicated as follows: *p < 0.05; **p < 0.01.

Together, the results above demonstrate tissue-specific differences between tissues in the BCR repertoire, including broad trends that are noted across patients. However, given the distinct immune cell profiles observed in each of the identified patient groups (**Figure 2**), we reasoned that BCR signatures would also vary by group. To assess this, we first evaluated differences in BCR clonal structure between patient groups by comparing repertoire polarization of IgM and IgG in PBMC and tumor. In both IgM and IgG, Group 1 and Group 2 tumor repertoires were more polarized than Group 4 (ANOVA: IgM *p*=0.043; IgG *p*=0.005; **Figures 9A, B**). Notably, we observed that, across samples from each group, the top 10% of clones (by size) in tumor were predominantly represented by “enriched” genes, indicating that as expected these tumor-enriched genes were likely driving the group differences in the degree of clonal expansion (**Figures 9C, D**). Particularly within the IgG compartment, enriched genes made up over 99% of the largest clones in most group 1 (n=7/11) and 2 (n=5/7) patients (**Figure 9D**). However, despite differences in clonal expansion between groups, we did not observe significant differences in SHM profiles among this set of clones in each group (**Figures 9E, F**).

### 3.8 Tumor IgG subisotype repertoire features associate with immune cell profile variation between and within patient groups

**Figure 10:**
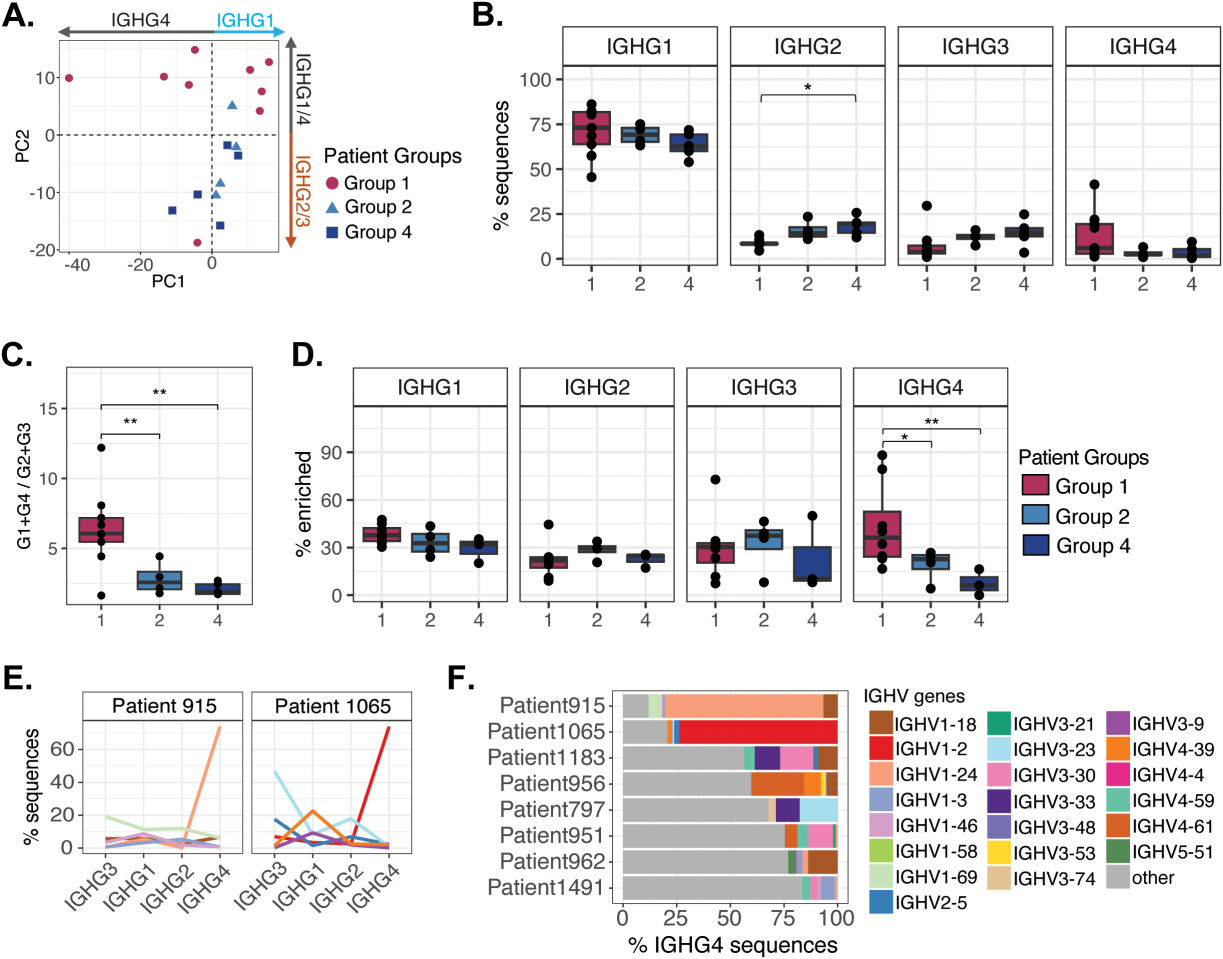
Characterization of tumor-infiltrating IgG subisotypes using FLAIRR-seq. **(A)** PCA of subisotype frequencies across patient groups (Group 1 n=9, Group 2 n=4, Group 4 n=5). **(B)** Boxplots illustrating the frequency distribution of subisotypes across patient groups, with inter-group comparisons conducted using unpaired t-tests. **(C)** Ratio of IGHG1+IGHG4 frequencies to IGHG2+IGHG3 compared between patient groups using unpaired t-tests. **(D)** Frequency of sequences with enriched IGHV genes per subisotype (Group 1, n=8; Group 2, n=4; Group 4, n=3). **(E)** Line plots showing usage frequency of most enriched IGHV genes across IgG subisotypes in two representative Group 1 patients. **(F)** Bar plots of the proportion of enriched IGHV gene usage in IGHG4 of Group 1 patients (n=8). Statistical significance was determined by paired t-test and indicated as follows: *p < 0.05; **p < 0.01; ***p < 0.001; ****p < 0.0001.

Our understanding of IgG subisotype variation in LUAD remains underexplored; however, it is well established that subisotypes can direct differential Ab-mediated immune responses. To expand on observations of bulk IgG repertoire profile differences between patient groups, we next leveraged a second higher-resolution AIRR-seq method that allowed us to characterize BCR repertoire features for each IgG subisotype in a subset of patients spanning Groups 1, 2, and 4 (n=18). This first revealed that Group 1 repertoires on average showed biased representation of IGHG1 and IGHG4 transcripts (**Figures 10A–C**), compared to Groups 2 and 4, which presented modest biases of IGHG2 and IGHG3. Additionally, we observed that a subset of patients within Group 1 uniquely showed elevated proportions of IGHG4 transcripts, in one instance exceeding 40% of the IgG repertoire (**Figure 10B**). Additionally, while we noted that tumor-enriched genes were found to represent large fractions of each subisotype, there were notable between-group differences in the representation of these genes among IGHG4 transcripts, revealing a particular elevation in Group 1 patients (**Figure 10D**). This was regardless of whether overall IGHG4 usage was elevated in a given patient. Notably, in two Group 1 patients, tumor-enriched IGHV genes comprised >75% of IGHG4 transcripts, dominated by a single gene that was uniquely elevated only in the IGHG4 compartment (**Figures 10E, F**).

**Figure 11:**
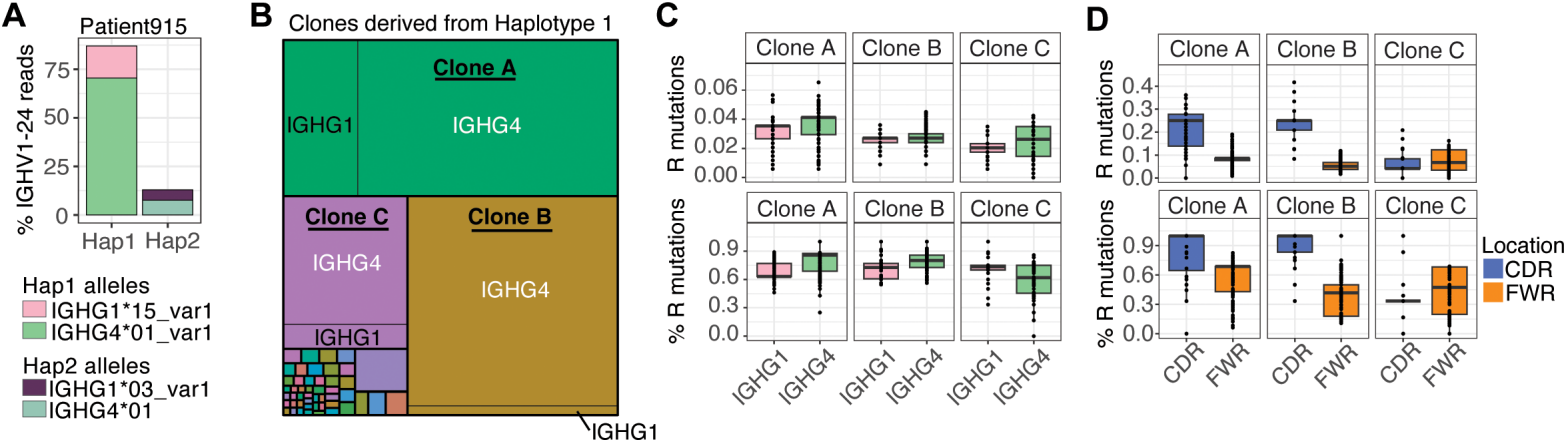
Group 1 tumors show selective IGHG4 class switch of enriched IGHV clones and coordinated TME features. **(A)** Total usage of IGHV1–24 in Patient 915, separated by haplotype and IGHG allele. **(B)** Treemap visualization of IGHV1–24 clones derived from haplotype 1 in Patient 915, with box size indicating clone frequency. **(C)** Replacement (R) mutation frequency (top) and percentage of R mutations (bottom) across the top three IGHV1–24 clones from haplotype 1, stratified by IGHG1 and IGHG4 alleles in haplotype 1. **(D)** Distribution of R mutations in CDR and FWR regions of top three IGHV1–24 clones in IGHG4 allele.

These results suggested that a subset of B cells in these patients had undergone class-switching to IGHG4 from IGHG1. Our use of FLAIRR-seq data allows us to gain resolution of IGHC gene allelic variation within the IgG repertoire, and thus link expressed VDJs to specific IGHC alleles. In the case of Patient 915, who was heterozygous for both IGHG1 and IGHG4 (**Supplementary Figure 14A**), we were able to link the dominant IGHV gene (IGHV1-24) to single IGHG1 and IGHG4 alleles determined to reside on the same chromosome (“Haplotype 1”; **Figure 11A**; **Supplementary Figure 14B**), providing direct evidence of class-switch recombination. We observed that the proportion of IGHG4-IGHV1–24 transcripts derived from this haplotype were 4.6-fold more abundant than those using the IGHG1 allele from the same haplotype. This was in contrast to IGHV1–24 transcripts expressed from Haplotype 2, which were far less abundant, and equally distributed between IGHG1 and IGHG4 (**Figure 11A**). IGHV1–24 transcripts from Haplotype 1 were represented by 51 clones, but dominated by three clones (**Figure 11B**), two of which shared the same IGHJ gene (IGHJ4), but distinct IGHD genes. In these three clones, IGHG4 transcripts were observed at a greater proportion, and in all cases showed higher amino acid replacement mutations than IGHG1 transcripts, and higher rates in CDRs than in FWRs (**Figures 11C, D**). Together, this analysis provides evidence that tumor-enriched genes in Patient 915 are overrepresented among IGHG4 transcripts, resulting from class-switch recombination and IGHG4-specific clonal expansion, and associated with increased SHM.

**Figure 12:**
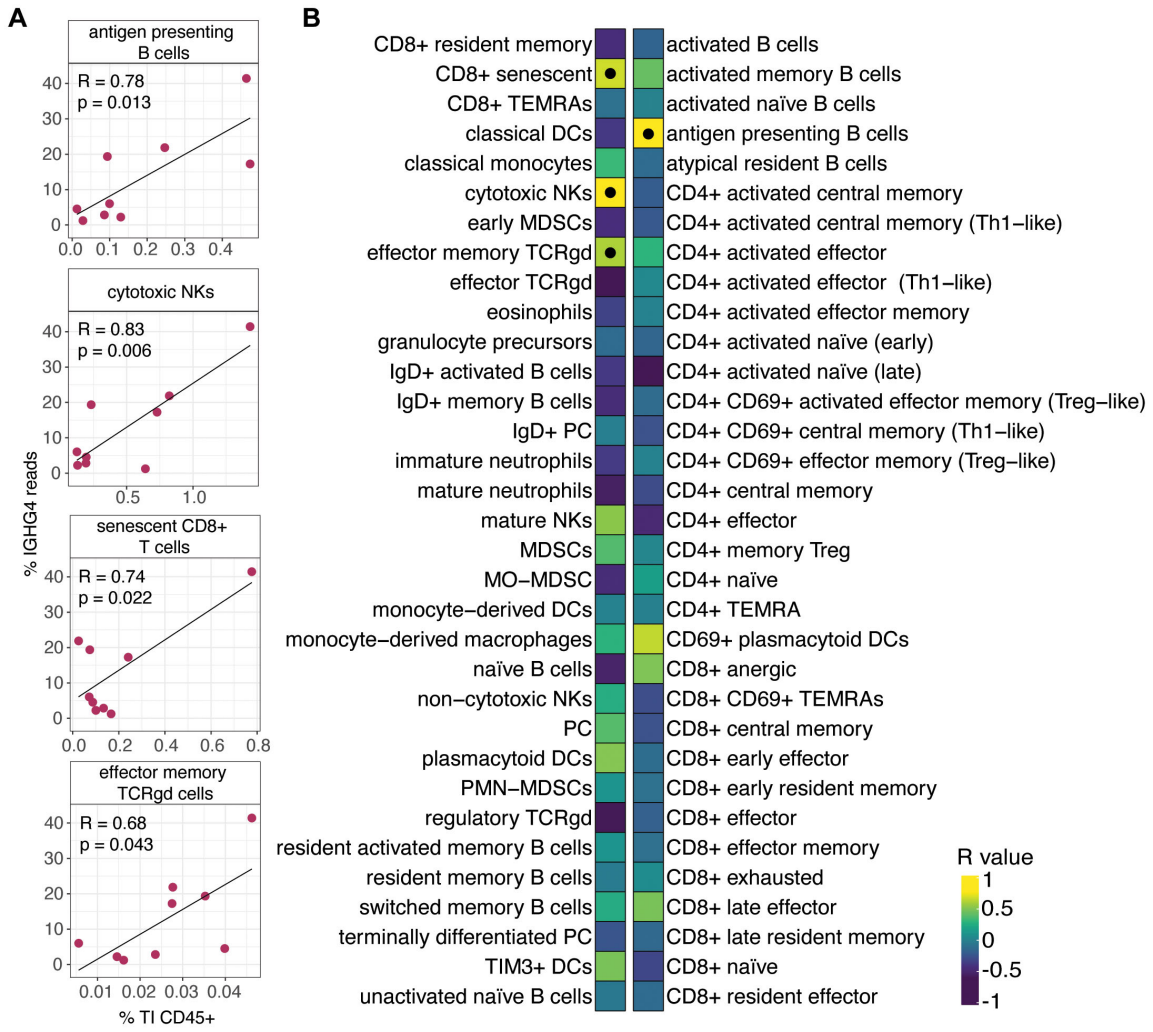
Correlations between IGHG4 usage and TI immune cell subsets in Group 1 patients. **(A)** Scatterplots showing representative correlations between IGHG4 sequence frequency and TI proportions of antigen presenting B cells, cytotoxic NK cells, effector memory TCRgd cells, and senescent CD8+ T cells (n=9). **(B)** Heatmap displaying correlation coefficients (R-values) across all TI immune cell subsets.

Although increased tumor-derived IgG4 is generally linked to immunosuppressive environments and poor prognosis in other solid cancers (40–42), we next asked whether IGHG4 frequency in Group 1 patients correlated with specific TI immune cell profiles. However, one study in LUAD found IgG4 could be positively associated with outcome depending on driver mutations, possibly due to its inability to form immune complexes (21). Other studies in LUAD have either not distinguished IgG subisotypes or omitted IgG4 altogether because of low abundance (10, 12). Here, we next asked whether IGHG4 frequency in Group 1 patients correlated with specific TI immune cell profiles. In our cohort, IGHG4 frequencies were positively correlated with four TI subsets: cytotoxic NK cells (R = 0.83, *p* = 0.059), antigen-presenting B cells (R = 0.78, *p* = 0.013), senescent CD8+ T cells (R = 0.74, *p* = 0.022), and effector memory TCRgd cells (R = 0.68, *p* = 0.043; **Figures 12A, B**). These results suggest that elevated IGHG4 may reflect coordinated changes in cytotoxic and antigen-presenting populations driven by local class-switching and clonal expansion, linking IGHG4 expression to shaping of the TI immune microenvironment. Notably, although not statistically significant, Group 1 patients with high IGHG4 shared a trend toward poorer overall survival when compared to other patient groups used in FLAIRR-seq as well as all other patient groups (**Supplementary Figures 15A, B**).

## 4 Discussion

In this study, we comprehensively characterized single-cell immune cell profiles in LUAD, analyzing peripheral blood, tumor tissue, and non-tumoral adjacent lung tissue from 48 treatment-naive patients. Expanding on previous studies focused on identifying trends in particular cell subsets, we leveraged the comprehensive nature of our dataset to more fully understand inter-patient variation in global immune profiles at high-resolution. This revealed that, while TME immune profiles across patients can show extensive variation, consistent signatures can be identified across patients that highlight unique features of their immune response potential. Given the established importance of TI B cells in LUAD, we focused on the detailed characterization of specific B cell subsets within these patient groups, pairing this cellular profiling data with analysis of the expressed BCR repertoire. This allowed us to: (i) extend earlier immune cell phenotyping, leading to higher-resolution descriptions of TI B cells alongside characterizations of additional adaptive and innate immune cells; and (ii) delineate key signatures from expressed BCRs within tumors, including clonal expansion dynamics, SHM, and biased IGHV, D, J and C gene usage. Together, these data provided evidence that TI B cells in a subset of patients are engaged in antigen-driven immune responses, but that critically, BCR signatures correlated with distinct immune cell profiles observed across identified patient groups, highlighting variable roles of B cells within the TME.

We identified 66 distinct immune cell clusters across tissues, noting significant tissue-specific differences in cell proportions, with 30 subsets elevated in tumor relative to peripheral blood, and 21 elevated in tumor compared to adjacent tissue. Specifically, we used an unsupervised clustering approach to identify four distinct patient groups characterized by unique tumor-infiltrating immune cell profiles. This allowed us to corroborate observations made by others in the literature, while also conducting more refined phenotyping of a greater number of cell subsets. Previous studies have shown that tumors enriched for mature or activated B cells often exhibit enhanced CD4+ T cell activation, Th1-like responses, and improved patient survival (8, 11, 19, 36). Alternatively, other LUAD studies have shown no significant differences in total TI B cell proportions while instead focusing on the density of T cell subsets and myeloid-derived cells associated with survival or response to immunotherapy (4, 10, 37). In our study, we have identified patient groups that likely mirror those identified previously. For example, Group 1 patients in our study were characterized by high proportions of various B cell subsets, including activated memory and antigen-presenting B cells, likely consistent with patients in other studies linking TI and TLS-associated B cells to improved outcomes (8, 11, 19, 36). Groups 2 and 3 were represented by T cell dominated signatures; specifically, group 2 exhibited enrichment of resident memory CD8+ T cells, whereas the TME of Group 3 patients was dominated by CD4+CD69+ effector T cells. In LUAD, both cytotoxic CD8+ T cells and certain CD4+ T cell subsets - particularly Th1 effector phenotypes in pro-inflammatory contexts - have also been linked to improved outcomes (8, 10, 11, 36, 37, 43). In contrast to Groups 1-3, Group 4 was dominated by myeloid cell populations (e.g., classical monocytes and early MDSCs) and naive and regulatory B and T cells, suggesting an immunologically inactive tumor environment. Similar profiles, marked by enrichment of monocytes, macrophages, and MDSCs, have been associated with impaired T cell activation and poor clinical outcomes, highlighting the immunosuppressive potential of myeloid-dominated TMEs (10, 44–46).

Building upon our initial observation of overall CD19+ B cell enrichment in Group 1 patients, we also assessed whether the distribution of B cell subpopulations within CD19+ cells was distinct among the four identified patient groups, hypothesizing that these differences could explain varying T cell profiles in the other patient groups. TI B cells in LUAD have been suggested to play roles via the production of tumor-specific antibodies, the presentation of antigen to CD4+ T cells, and the modulation of T cell responses toward either pro-inflammatory or regulatory phenotypes (8, 10, 11, 19). Out of 15 identified B cell clusters, five showed statistically significant variation among the patient groups: activated memory B cells, IgD+ memory B cells, inflammatory B cells, Bregs, and antigen-presenting B cells. Activated memory B cells were the most frequent B cell subset across all groups, although this subset was lowest in Group 4. Group 4 was indeed the most distinct, with lower proportions of activated memory and antigen-presenting B cells, but significantly higher proportions of IgD+ memory, inflammatory B cells, and Bregs compared to Group 1. These findings demonstrate that not only were B cells depleted in group 4, but also that the observed proportions of TI B cell subpopulations were likely indicative of the absence of an active humoral response. In Group 2, the combination of fewer antigen-presenting B cells and more resident effector memory CD4+ T cells suggests that B cell-mediated priming may have already occurred, consistent with NSCLC reports where Th1-skewed CD4+ T cells and cytotoxic CD8+ T cell signatures predict better prognosis, even without differences in overall B cell proportions (37). Group 3 patients were among those with the highest proportion of activated memory B cells alongside a notably high proportion of CD69+ effector memory CD4+ T cells, suggesting B cells are supporting the CD4+ T cell response as noted in previous studies (8, 11, 19). Group 1, in contrast, maintained consistently high proportions of multiple B cell subsets, including activated memory and antigen presenting B cells, suggesting an active and sustained humoral response within the TME. These B cell subsets may be simultaneously generating tumor-specific antibodies, presenting antigen to CD4+ T cells, and shaping pro-inflammatory T cell responses, therefore sustaining a coordinated antitumor immune response in line with previous reports of TI and TLS-associated B cells contributing to improved LUAD outcomes.

The Ab response is fundamentally linked to B cell function and activity. To our knowledge, our study is the first to comprehensively profile expressed bulk BCR repertoires in matched tissues within LUAD patients to identify tumor-specific characteristics. Most notably, BCR repertoires in LUAD tumors and adjacent tissues showed increased clonal expansion and reduced diversity compared to peripheral blood. This was accompanied by higher SHM in tumor IgM compared to PBMC and non-tumor tissue, and in increased fractions of replacement mutations in both IgM and IgG. Smaller studies have also observed high levels of mutation in LUAD tumor BCRs (12, 24, 47). It is notable that in the periphery, as expected, IgM showed low levels of SHM, which we reasoned reflected the fact that expressed IgM BCRs in peripheral blood were likely the products of naive B cells. This was in contrast to relatively extensive SHM in IgM observed in tumor tissue. However, because BCRs in our study were amplified in bulk from tissue-isolated RNA, we cannot directly link tumor IgMs to specific cell subsets; in future studies, these cell-to-isotype links will be critical to define via single cell BCR sequencing to more fully resolve the potential roles of IgM Abs in tumor. Nonetheless, these observed signatures were indicative of affinity maturation and that tumor-infiltrating BCRs played a role in antigen recognition. Supporting this, it has been shown previously that IgM, IgG, and IgA Abs from LUAD tumor isolates were capable of binding known cancer antigens (19, 48, 49).

Extending this analysis, we identified IGHV genes enriched in tumor IgM and IgG repertoires compared to PBMC. These enriched genes displayed higher SHM, larger clone sizes, and distinct CDR3 features, consistent with tumor-associated selection. IgM enrichment patterns were relatively consistent across patients compared to IgG, in which enriched genes tended to show common patterns only among subsets of patients. In nearly all cases, tumor-enriched IGHV genes were linked to clonal expansion and affinity maturation, indicating an active, productive humoral response within the TME. A previous study reported that early recurrence was associated with increased usage of specific IGHV and IGHJ genes, but these were not evaluated for clonal expansion, somatic hypermutation, or isotype context (23). Whether these recurrence-associated genes reflect similar selective pressures is unclear, but our isotype-resolved analysis demonstrates that tumor-enriched IGHV usage is tightly linked to clonal expansion and affinity maturation, with distinct enrichment patterns between IgM and IgG repertoires. These findings underscore the added value of isotype- and patient-specific analyses in defining the functional dynamics of TI B cells.

Finally, we were able to link IgG tumor-enriched genes to differential patterns in IgG subisotype usage among the patients of Groups 1, 2, and 4. Specifically, we found that Group 1 patients, on average, showed biased usage of IGHG1 and IGHG4 transcripts relative to Groups 2 and 4. Notably, a subset of Group 1 patients exhibited uniquely high proportions of IGHG4, in some cases exceeding 40% of their total IgG repertoire. Furthermore, we found that tumor-enriched genes were uniquely elevated in IGHG4 transcripts of Group 1 patients regardless of the overall IGHG4 usage. In two Group 1 patients, specific tumor-enriched IGHV genes dominated over 75% of IGHG4 transcripts, providing evidence for class-switching from IGHG1 to IGHG4 in a subset of B cells. Direct evidence of this was obtained from Patient 915, who was heterozygous for both IGHG1 and IGHG4, in which we observed IGHG4 allele-specific clonal expansion, and associated increases in amino acid replacement mutations in CDR regions, consistent with increased somatic hypermutation after class-switching. These patterns suggest that Group 1 patient B cells are experiencing chronic antigen exposure and a cytokine milieu that favors IgG4 class switching, driven by the accompanying TME.

Overall, the functional implications of increased IGHG4 remain biologically complex as it posesses several structural features that generally limit classical effector functions and are commonly associated with regultory functions or immunosuppression (21, 40–42). Notably, IGHG4 enrichment in solid tumors often correlates with poorer outcomes. Thus, the elevated IGHG4 in Group 1 patients could reflect aspects of an exhausted or tolerogenic environment rather than enhanced anti-tumor functions.

At the same time, IGHG4 biology is not exclusively suppressive. A previous LUAD study reported a driver mutation-context dependent positive association potentially due to IgG4’s limited immune-complex formation (21). Other experimental work has shown that IGHG4 can activate complement under specific biochemical or structural conditions, although such mechanisms have not been demonstrated in LUAD (21, 40–42). Collectively, these findings underscore that IGHG4 function is highly context-dependent and remains incompletely understood.

In our cohort, we found that increased IGHG4 frequencies positively correlated with cytotoxic NK cells, antigen-presenting B cells, senescent CD8+ T cells, and effector memory TCRgd cells, supporting a model in which localized switching and IGHG4-focused clonal expansion can emerge in lymphocyte-dominant TMEs. Notably, a recent study in NSCLC identified a plasma cell/NK cell tumor profile that was linked to better survival despite low expression of canonical response genes (50). Complementing this, some experimental work shows NK cells can amplify cytokine circuits that influence class-switch recombination, enhance B cell antigen processing and presentation, and shape memory T cell development (51). However, when incorporating clinical data, we found that the four Group 1 patients with high IGHG4 usage displayed overall shorter survival compared to other Group 1 patients and Groups 2-4 (**Supplementary Figure 15**). These differences were not statistically significant in global or pairwise log-rank tests, likely reflecting the small sample size, but the consistent directional trend suggests that elevated IGHG4 may mark a distinct subset within Group 1 characterized by different immune pressures or functional states.

Together, these results indicate that IGHG4 enrichment represents a biologically heterogeneous signal. Although IGHG4 enriched Group 1 tumors share features of suggestive coordinated anti-tumor responses, the survival trend we observed suggests that this same signature may also mark patients with a dysregulated humoral response. Thus, IGHG4 bias in LUAD should not be viewed as a straightforward surrogate of either enhanced or suppressed immunity. More broadly, our findings underscore that patient stratification based solely on bulk immune cell proportions may obscure meaningful functional and repertoire-level differences. Integrating B cell subset composition, class switch patterns, and IgG subisotype architecture provides a more accurate framework for understanding tumor immunity and may prevent overly simplistic or potentially inaccurate assumptions when predicting clinical outcomes and treatment options.

These repertoire differences parallel distinct TME profiles. Group 1 combines high B cell infiltration with abundant activated memory and antigen-presenting B cells, alongside cytotoxic and effector T cell populations. The IGHG4 enrichment in this group, supported by haplotype and clonal analyses showing local class-switching and affinity maturation, likely reflects a dysregulated humoral response. Group 2, despite strong clonal expansion, has lower total B cell and antigen-presenting B cell abundance, suggesting active humoral selection but more limited T cell engagement; its higher IGHG2/IGHG3 usage may represent a less skewed, less integrated immune response. Group 4 shows minimal B cell expansion, a predominance of myeloid and regulatory cells, and IgG subisotype patterns consistent with an immunologically inactive TME.

These findings emphasize that B cell subset composition, BCR clonal expansion dynamics, and IgG subisotype biases represent promising biomarkers for refining LUAD patient stratification. By grouping patients according to the proportional composition of all TI immune cell subsets, we identified four immune-defined groups that separate patients who would otherwise be collapsed into a binary “activated” versus “suppressive” classification model. The added resolution provided by BCR repertoire features and resolving IgG subisotype distributions further supports the view that LUAD immune landscapes exist along a continuum rather than two discrete states. Incorporating these humoral features into TME profiling frameworks may help identify patients with robust B cell mediated engagement versus those with minimally active or dysregulated B cell compartments. Such distinctions could inform the rational design or selection of antibody-based therapies and guide tailored immunotherapeutic strategies that align with the dominant humoral architecture of individual tumors.

## Data Availability

The CyTOF dataset is not readily available because the mandatory cytometry repository (FlowReponsitory) is currently under a temporary suspension of new experiment creation, which prevents deposition of new datasets. Processed CyTOF data have been deposited in ImmPort under accession number SDY3293 and will be made public at the next release date (01/29/2026). Requests to access the raw CyTOF data can be directed C.T.W. or K.M.R. The AIRR-seq and FLAIRR-seq datasets generated in this study are publicly availabe in the NCBI Sequence Read Archive (SRA) as part of BioProject PRJNA137180. The data can be found here: https://www.ncbi.nlm.nih.gov/sra/?term=PRJNA1374180.

## References

[1] American Cancer Society . What is lung cancer. Atlanta (GA): American Cancer Society (2024). Available online at: https://www.cancer.org/cancer/types/lung-cancer/about/what-is.html.

[2] American Cancer Society . Immunotherapy. Atlanta (GA): American Cancer Society (2025). Available online at: https://www.cancer.net/navigating-cancer-care/how-cancer-treated/immunotherapy-and-vaccines/what-immunotherapy.

[3] Sautès-Fridman C Petitprez F Calderaro J Fridman WH . Tertiary lymphoid structures in the era of cancer immunotherapy. Nat Rev Cancer. (2019) 19:307–25., PMID: 31092904 10.1038/s41568-019-0144-6

[4] Duchemann B Naigeon M Auclin E Ferrara R Cassard L Jouniaux JM . CD8+PD-1+ to CD4+PD-1+ ratio (PERLS) is associated with prognosis of patients with advanced NSCLC treated with PD-(L)1 blockers. J Immunother Cancer. (2022) 10:e004012. doi: 10.1136/jitc-2021-004012, PMID: 35131864 PMC8823243

[5] Helmink BA Reddy SM Gao J Zhang S Basar R Thakur R . B cells and tertiary lymphoid structures promote immunotherapy response. Nature. (2020) 577:549–55. doi: 10.1038/s41586-019-1922-8, PMID: 31942075 PMC8762581

[6] Wu H Chen C Gu L Li J Yue Y Lyu M . B cell deficiency promotes the initiation and progression of lung cancer. Front Oncol. (2022) 12:5336. doi: 10.3389/fonc.2022.1006477, PMID: 36249034 PMC9556970

[7] Leong TL . Bryant VL. B Cells Lung cancer—not just bystander cell: literature review. Transl Lung Cancer Res. (2021) 10:2830–41. doi: 10.21037/tlcr-20-788, PMID: 34295681 PMC8264333

[8] Bruno TC Ebner PJ Moore BL Squalls OG Waugh KA Eruslanov EB . Antigen-presenting intratumoral B cells affect CD4+ TIL phenotypes in non–small cell lung cancer patients. Cancer Immunol Res. (2017) 5:898–907. doi: 10.1158/2326-6066.CIR-17-0075, PMID: 28848053 PMC5788174

[9] Laumont CM Banville AC Gilardi M Hollern DP Nelson BH . Tumour-infiltrating B cells: immunological mechanisms, clinical impact and therapeutic opportunities. Nat Rev Cancer. (2022) 22:414–30. doi: 10.1038/S41568-022-00466-1, PMID: 35393541 PMC9678336

[10] Leader AM Grout JA Maier BB Nabet BY Park MD Tabachnikova A . Single-cell analysis of human non-small cell lung cancer lesions refines tumor classification and patient stratification. Cancer Cell. (2021) 39:1594–1609.e12. doi: 10.1016/j.ccell.2021.10.009, PMID: 34767762 PMC8728963

[11] Germain C Devi-Marulkar P Knockaert S Biton J Kaplon H Letaïef L . Tertiary lymphoid structure B cells narrow regulatory T cells impact in lung cancer patients. Front Immunol. (2021) 12:626776. doi: 10.3389/fimmu.2021.626776, PMID: 33763071 PMC7983944

[12] Hao D Han G Sinjab A Gomez-Bolanos LI Lazcano R Serrano A . The single-cell immunogenomic landscape of B and plasma cells in early-stage lung adenocarcinoma. Cancer Discovery. (2022) 12:2626–44. doi: 10.1158/2159-8290.CD-21-1658, PMID: 36098652 PMC9633381

[13] Patel AJ Khan N Richter A Naidu B Drayson MT Middleton GW . Deep immune B and plasma cell repertoire in non-small cell lung cancer. Front Immunol. (2023) 14:1198665. doi: 10.3389/fimmu.2023.1198665, PMID: 37398676 PMC10311499

[14] Rossetti RAM Lorenzi NPC Yokochi K Rosa MBSF Benevides L Margarido PFR . B lymphocytes can be activated to act as antigen-presenting cells to promote anti-tumor responses. PloS One. (2018) 13:e0199034. doi: 10.1371/journal.pone.0199034, PMID: 29975708 PMC6033398

[15] Sharonov GV Serebrovskaya EO Yuzhakova DV Britanova OV . Chudakov DM. B cells plasma Cells antibody repertoires tumour microenvironment. Nat Rev Immunol. (2020) 20:294–307. doi: 10.1038/s41577-019-0257-x, PMID: 31988391

[16] Yuen GJ Demissie E Pillai S . and cancer: a love-hate relationship. Trends Cancer. (2016) 2:747–57. doi: 10.1016/j.trecan.2016.10.010, PMID: 28626801 PMC5472356

[17] Deola S Panelli MC Maric D Selleri S Dmitrieva NI Voss CY . Helper B cells promote cytotoxic T cell survival and proliferation independently of antigen presentation through CD27/CD70 interactions. J Immunol. (2008) 180:1362–72. doi: 10.4049/jimmunol.180.3.1362, PMID: 18209030

[18] Song L Ouyang Z Cohen D Cao Y Altreuter J Bai G . Comprehensive characterizations of immune receptor repertoire in tumors and cancer immunotherapy studies. Cancer Immunol Res. (2022) 10:788–99. doi: 10.1158/2326-6066.CIR-21-0965, PMID: 35605261 PMC9299271

[19] Germain C Gnjatic S Tamzalit F Knockaert S Remark R Goc J . Presence of B cells in tertiary lymphoid structures is associated with a protective immunity in patients with lung cancer. Am J Respir Crit Care Med. (2014) 189:832–44. doi: 10.1164/rccm.201309-1611OC, PMID: 24484236

[20] James LK . B cells defined by immunoglobulin isotypes. Clin Exp Immunol. (2022) 210:230–9. doi: 10.1093/cei/uxac091, PMID: 36197112 PMC9985177

[21] Isaeva OI Sharonov GV Serebrovskaya EO Turchaninova MA Zaretsky AR Shugay M . Intratumoral immunoglobulin isotypes predict survival in lung adenocarcinoma subtypes. J Immunother Cancer. (2019) 7:279. doi: 10.1186/s40425-019-0747-1, PMID: 31665076 PMC6819482

[22] Su H Wang Y Khan S Huang Y Yi Z Zhu N . Immunotherapy shapes B-cell receptor repertoire to induce anti-tumor antibodies production in colon and lung cancer. Genome Instab Dis. (2024) 5:183–96. doi: 10.1007/s42764-024-00134-8

[23] Liu J Yang X Lu X Zhang L Luo W Cheng Y . Impact of T-cell receptor and B-cell receptor repertoire on the recurrence of early-stage lung adenocarcinoma. Exp Cell Res. (2020) 394:112134. doi: 10.1016/j.yexcr.2020.112134, PMID: 32540399

[24] DeFalco J Harbell M Manning-Bog A Baia G Scholz A Millare B . Non-progressing cancer patients have persistent B cell responses expressing shared antibody paratopes that target public tumor antigens. Clin Immunol. (2018) 187:37–45. doi: 10.1016/j.clim.2017.10.002, PMID: 29031828

[25] Nowicka M Krieg C Crowell HL Weber LM Hartmann FJ Guglietta S . CyTOF workflow: differential discovery in high-throughput high-dimensional cytometry datasets. F1000Res. (2017) 6:748. doi: 10.12688/f1000research.11622.3, PMID: 28663787 PMC5473464

[26] Wilkerson MD Hayes DN . ConsensusClusterPlus: a class discovery tool with confidence assessments and item tracking. Bioinformatics. (2010) 26:1572–3. doi: 10.1093/bioinformatics/btq170, PMID: 20427518 PMC2881355

[27] Finak G Jiang W Gottardo R . CytoML for cross-platform cytometry data sharing. Cytometry A. (2018) 93:1189–95. doi: 10.1002/cyto.a.v93.12, PMID: 30551257 PMC6443375

[28] Ford EE Tieri D Rodriguez OL Francoeur NJ Soto J Kos JT . FLAIRR-Seq: a method for single-molecule resolution of near full-length antibody H chain repertoires. J Immunol. (2023) 210:1607–19. doi: 10.4049/jimmunol.2200825, PMID: 37027017 PMC10152037

[29] Gupta NT Vander Heiden JA Uduman M Gadala-Maria D Yaari G Kleinstein SH . Change-O: a toolkit for analyzing large-scale B cell immunoglobulin repertoire sequencing data. Bioinformatics. (2015) 31:3356–8. doi: 10.1093/bioinformatics/btv359, PMID: 26069265 PMC4793929

[30] Vander Heiden JA Yaari G Uduman M Stern JNH O’Connor KC Hafler DA . pRESTO: a toolkit for processing high-throughput sequencing raw reads of lymphocyte receptor repertoires. Bioinformatics. (2014) 30:1930–2. doi: 10.1093/BIOINFORMATICS/BTU138, PMID: 24618469 PMC4071206

[31] Li H . Minimap2: pairwise alignment for nucleotide sequences. Bioinformatics. (2018) 34:3094–100. doi: 10.1093/BIOINFORMATICS/BTY191, PMID: 29750242 PMC6137996

[32] Li H Handsaker B Wysoker A Fennell T Ruan J Homer N . The sequence alignment/map format and SAMtools. Bioinformatics. (2009) 25:2078–9. doi: 10.1093/BIOINFORMATICS/BTP352, PMID: 19505943 PMC2723002

[33] Martin M Patterson M Garg S Fischer SO Pisanti N Klau GW . WhatsHap: fast and accurate read-based phasing. bioRxiv. 2016:085050. doi: 10.1101/085050

[34] Fu L Niu B Zhu Z Wu S Li W . CD-HIT: accelerated for clustering the next-generation sequencing data. Bioinformatics. (2012) 28:3150–2. doi: 10.1093/bioinformatics/bts565, PMID: 23060610 PMC3516142

[35] Altschul SF Gish W Miller W Myers EW Lipman DJ . Basic local alignment search tool. J Mol Biol. (1990) 215:403–10. doi: 10.1016/S0022-2836(05)80360-2, PMID: 2231712

[36] Dieu-Nosjean MC Antoine M Danel C Heudes D Wislez M Poulot V . Long-term survival for patients with non-small-cell lung cancer with intratumoral lymphoid structures. J Clin Oncol. (2008) 26:4410–7. doi: 10.1200/JCO.2007.15.0284, PMID: 18802153

[37] Goc J Germain C Vo-Bourgais TKD Lupo A Klein C Knockaert S . Dendritic cells in tumor-associated tertiary lymphoid structures signal a Th1 cytotoxic immune contexture and license the positive prognostic value of infiltrating CD8+ T cells. Cancer Res. (2014) 74:705–15. doi: 10.1158/0008-5472.CAN-13-1342, PMID: 24366885

[38] Budczies J Kirchner M Kluck K Kazdal D Glade J Allgäuer M . A gene expression signature associated with B cells predicts benefit from immune checkpoint blockade in lung adenocarcinoma. Oncoimmunology. (2021) 10:1860586. doi: 10.1080/2162402X.2020.1860586, PMID: 33520406 PMC7808386

[39] Aizik L Dror Y Taussig D Barzel A Carmi Y Wine Y . Antibody repertoire analysis of tumor-infiltrating B cells reveals distinct signatures and distributions across tissues. Front Immunol. (2021) 12:2820. doi: 10.3389/fimmu.2021.705381, PMID: 34349765 PMC8327180

[40] Wang H Li J Wang Y Chen Y Zhang W Pan X . IgG4-mediated M2 macrophage polarization in tertiary lymphoid structures of esophageal cancer: implications for immunosuppression. Front Immunol. (2024) 15:1497783. doi: 10.3389/fimmu.2024.1497783, PMID: 39896813 PMC11782137

[41] Karagiannis P Gilbert AE Josephs DH Ali N Dodev T Saul L . IgG4 subclass antibodies impair antitumor immunity in melanoma. J Clin Invest. (2013) 123:1457–74. doi: 10.1172/JCI65579, PMID: 23454746 PMC3613918

[42] Wang H Xu Q Zhao C Zhu Z Zhu X Zhou J . An immune evasion mechanism with IgG4 playing an essential role in cancer and implication for immunotherapy. J Immunother Cancer. (2020) 8:e000661. doi: 10.1136/jitc-2020-000661, PMID: 32819973 PMC7443307

[43] Ganesan AP Clarke J Wood O Garrido-Martin EM Chee SJ Mellows T . Tissue-resident memory features are linked to the magnitude of cytotoxic T cell responses in human lung cancer. Nat Immunol. (2017) 18:940–50. doi: 10.1038/ni.3775, PMID: 28628092 PMC6036910

[44] Thorsson V Gibbs DL Brown SD Wolf D Bortone DS Ou Yang TH . The immune landscape of cancer. Immunity. (2018) 48:812–830.e14. doi: 10.1016/j.immuni.2018.03.023, PMID: 29628290 PMC5982584

[45] Sangaletti S Ferrara R Tripodo C Garassino MC Colombo MP . Myeloid cell heterogeneity in lung cancer: implication for immunotherapy. Cancer Immunol Immunother. (2021) 70:2429–40. doi: 10.1007/S00262-021-02916-5, PMID: 33797567 PMC8017108

[46] Veglia F Sanseviero E Gabrilovich DI . Myeloid-derived suppressor cells in the era of increasing myeloid cell diversity. Nat Rev Immunol. (2021) 21:485–98. doi: 10.1038/s41577-020-00490-y, PMID: 33526920 PMC7849958

[47] Krasik SV Bryushkova EA Sharonov GV Myalik DS Shurganova EV Komarov DV . Systematic evaluation of intratumoral and peripheral BCR repertoires in three cancers. Elife. (2025) 13. doi: 10.7554/ELIFE.89506, PMID: 39831798 PMC11745494

[48] Imahayashi S Ichiyoshi Y Yoshino I Eifuku R Takenoyama M Yasumoto K . Tumor-infiltrating B-cell-derived IgG recognizes tumor components in human lung cancer. Cancer Invest. (2000) 18:530–6. doi: 10.3109/07357900009012192, PMID: 10923101

[49] Zhang X Li J Wang Y Liu M Liu F Zhang X . A diagnostic model with IgM autoantibodies and carcinoembryonic antigen for early detection of lung adenocarcinoma. Front Immunol. (2022) 12:728853. doi: 10.3389/fimmu.2021.728853, PMID: 35140701 PMC8818794

[50] Backman M La Fleur L Kurppa P Djureinovic D Elfving H Brunnström H . Infiltration of NK and plasma cells is associated with a distinct immune subset in non-small cell lung cancer. J Pathol. (2021) 255:243–56. doi: 10.1002/path.v255.3, PMID: 34339045

[51] Jennings P Yuan D . NK cell enhancement of antigen presentation by B lymphocytes. J Immunol. (2009) 182:2879–87. doi: 10.4049/jimmunol.0803220, PMID: 19234183

