# Supplementary material for: B cell phenotypes and antibody signatures associate with interpatient variation in the lung adenocarcinoma tumor microenvironment
Source: Front Immunol. 2026 Jan 28;16:1739637. doi: 10.3389/fimmu.2025.1739637 (PMC12891238; doi:10.3389/fimmu.2025.1739637)
Supplement: Supplementary file 1 [file DataSheet1.docx]

**Supplementary Material**


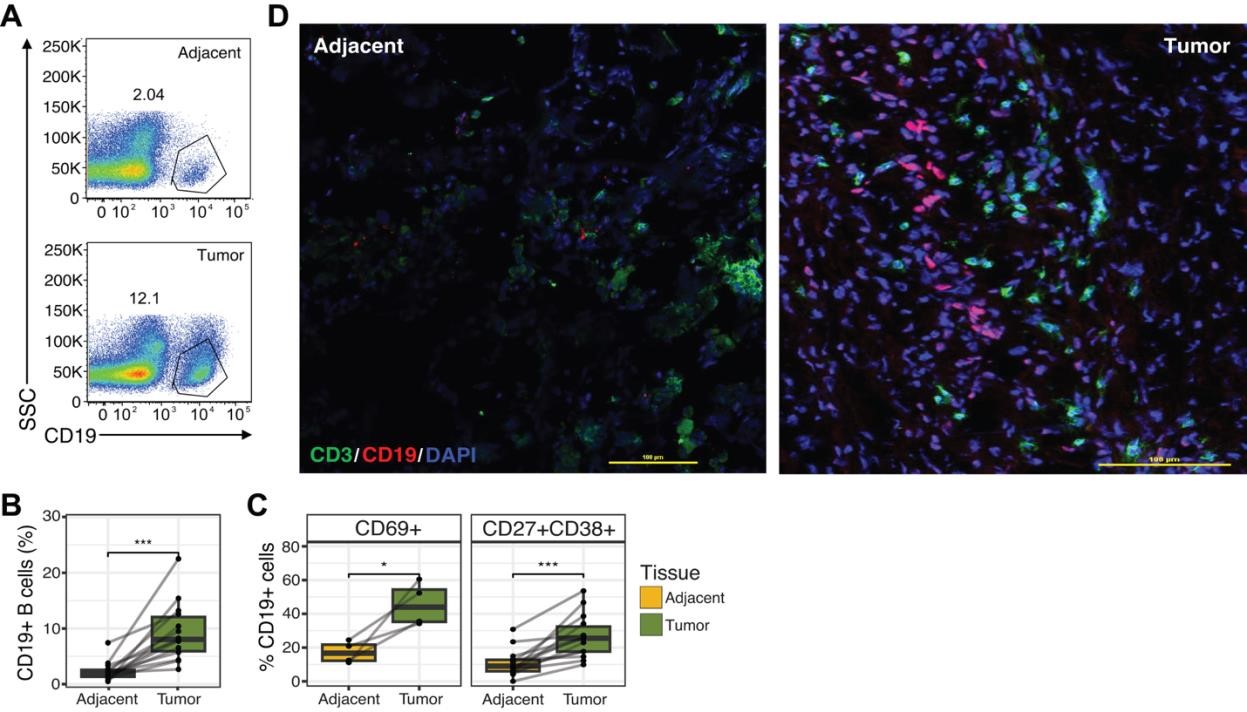


**Supp. Fig. 1. Increased density and altered subsets of B cells in LUAD tumors.** (**A & B**) Flow cytometry showing a higher frequency of CD19⁺ B cells in tumor versus matched adjacent lung tissue (n=22 patients; paired t-test, p=8.02×10⁻⁵). (**C**) Tumor-enriched B cell subsets (CD19⁺CD27⁺CD38⁺ and CD19⁺CD69⁺) relative to adjacent tissue (paired t-tests; p-values indicated). (**D**) Representative confocal images from one patient showing tumor and adjacent tissue showing colocalization of CD3⁺ T cells and CD19⁺ B cells.


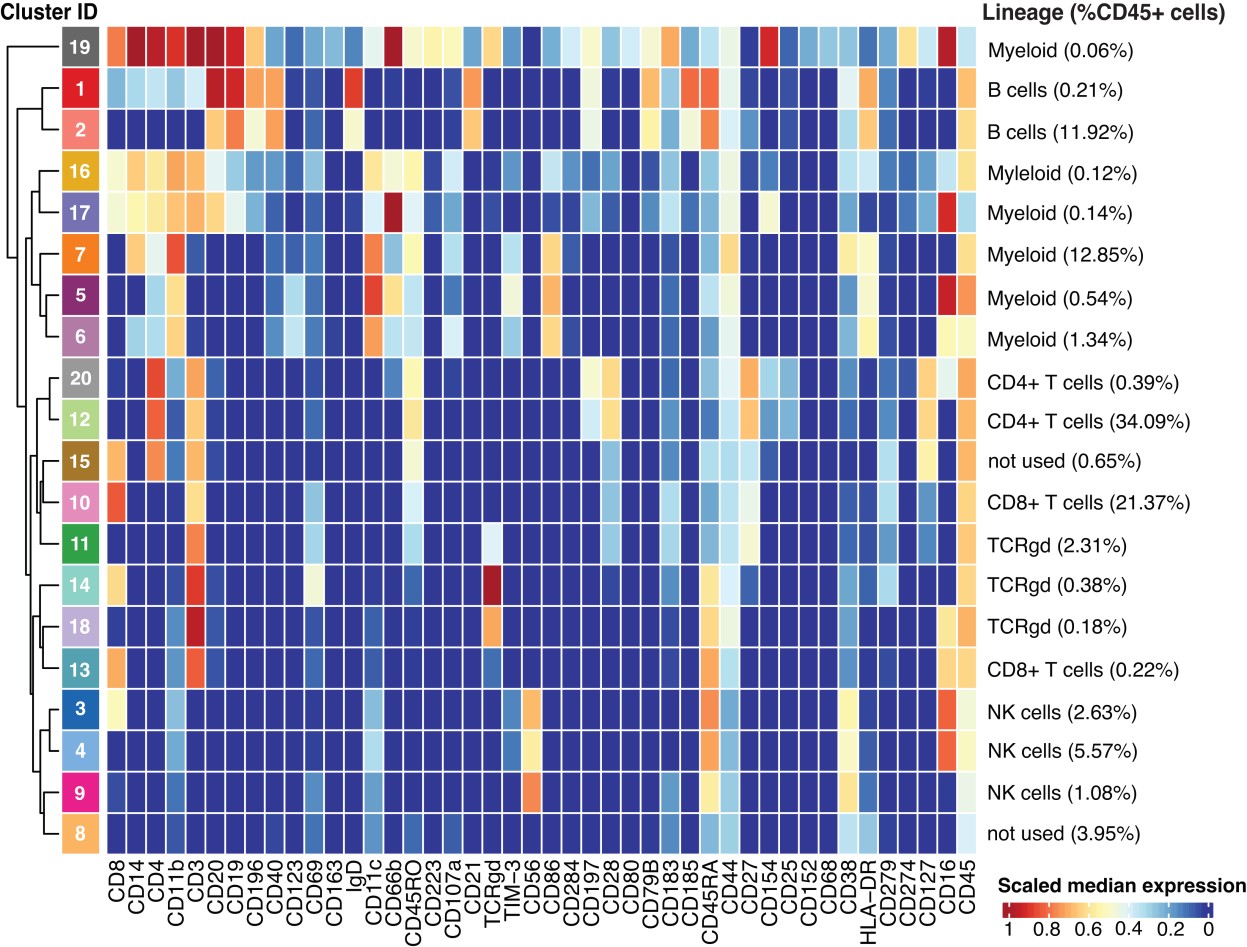


**Supp. Fig. 2. Lineage heatmap of CD45+ cells.** CyTOF analysis was performed using a 44marker panel to profile immune populations across tissue samples. CD45⁺ events were clustered based on expression of canonical lineage markers (Table 1), resolving 20 immune populations representing both lymphoid and myeloid lineages. Heatmap shows relative marker expression used to define CD3⁺CD8⁺ (2 clusters), CD3⁺CD4⁺ (2), CD3⁻CD19⁺ B cells (2), CD16⁺CD56⁺CD3⁻CD19⁻ NK cells (3), CD3⁺TCRgd⁺ T cells (3), and CD11b⁺ myeloid populations.


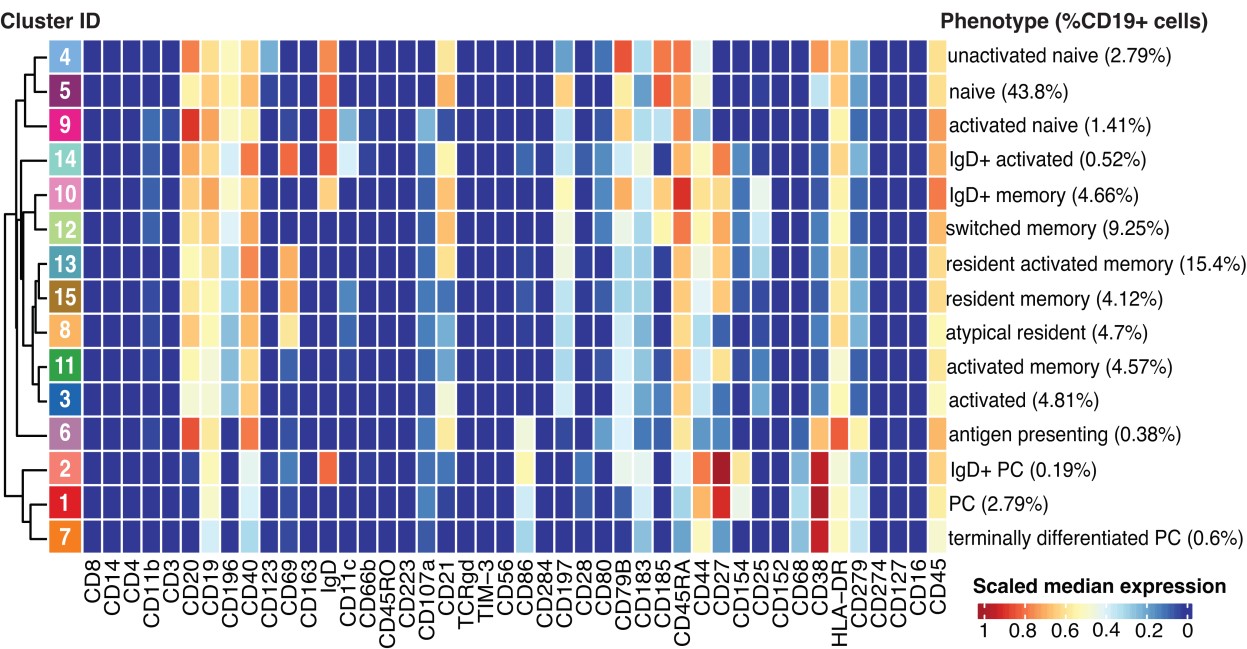


**Supp. Fig. 3: Lineage heatmap of CD19+ cells.** CD3-CD19+ B cell clusters from Supp. Fig. 1 were combined and re-clustered based on relative expression of canonical B cell lineage markers (Table 2). Phenotypes for each cluster were inferred using expression of lineage markers and additional functional markers.


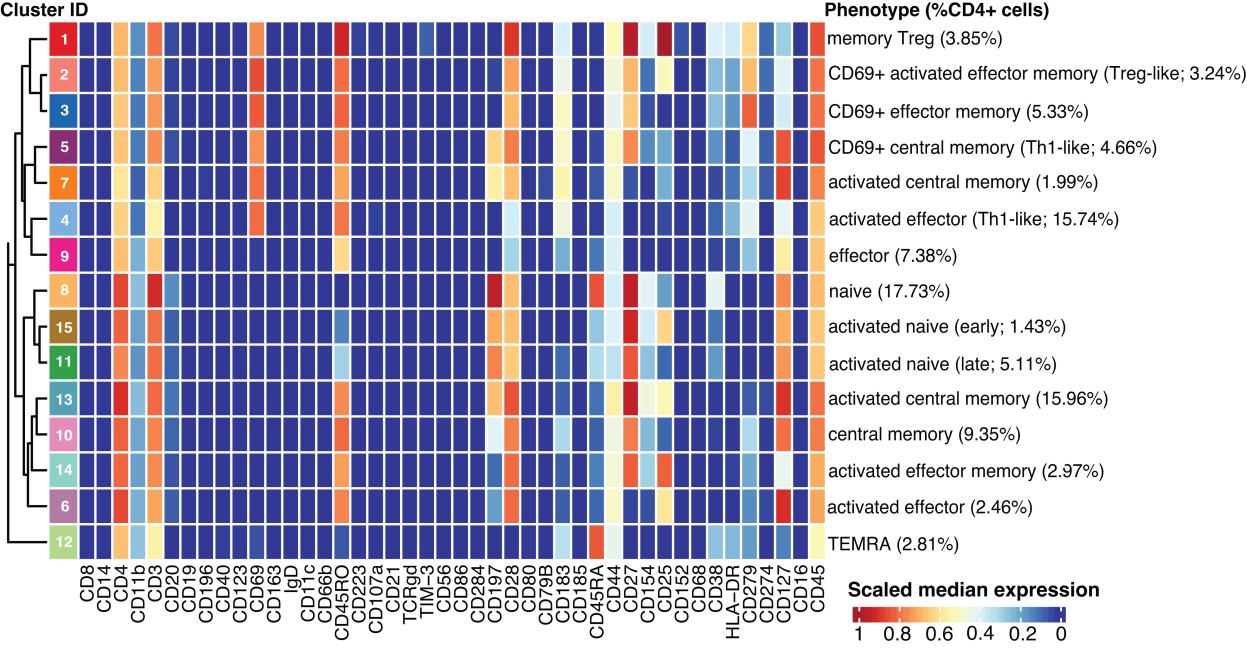


**Supp. Fig. 4. Lineage heatmap of CD4+ cells.** CD3+CD4+ T cell clusters from Supp. Fig. 1

were combined and re-clustered based on relative expression of canonical T cell lineage markers (Table 2). Phenotypes for each cluster were inferred using expression of lineage markers and additional functional markers.


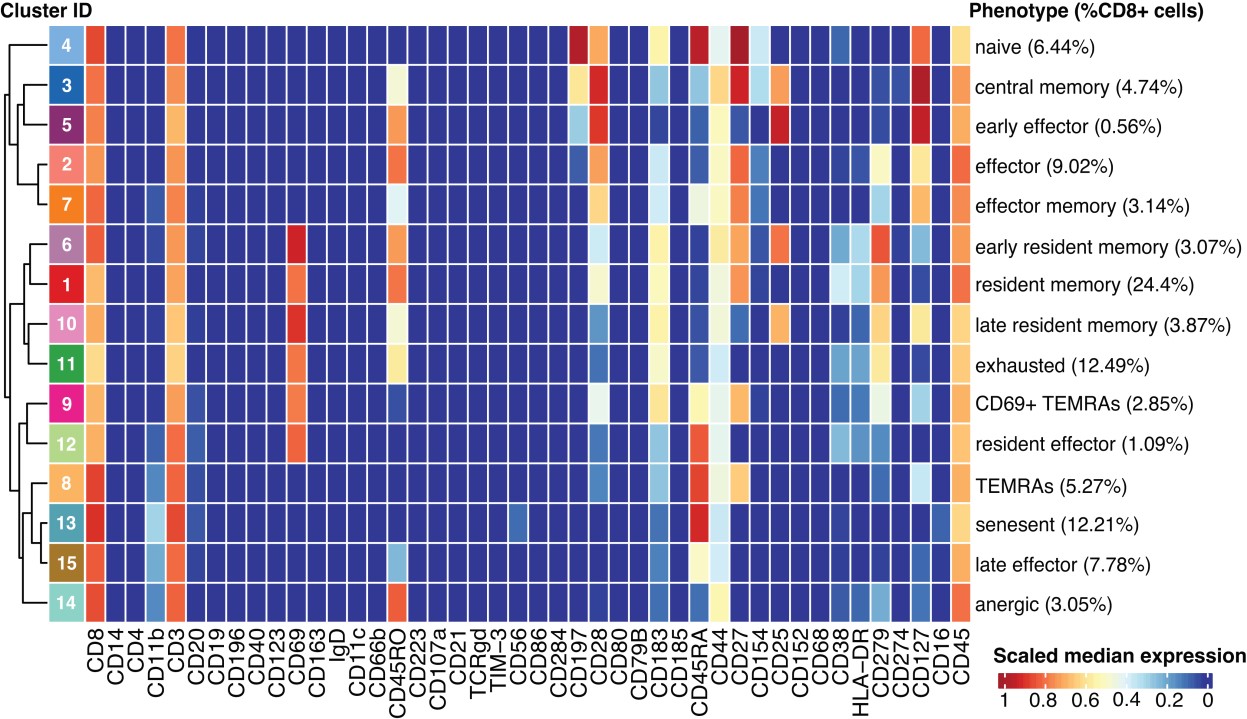


# Supp. Fig. 5: Lineage heatmap of CD8+ cells

CD3+CD8+ T cell clusters from Supp. Fig. 1 were combined and re-clustered based on relative expression of canonical T cell lineage markers (Table 2). Phenotypes for each cluster were inferred using expression of lineage markers and additional functional markers.


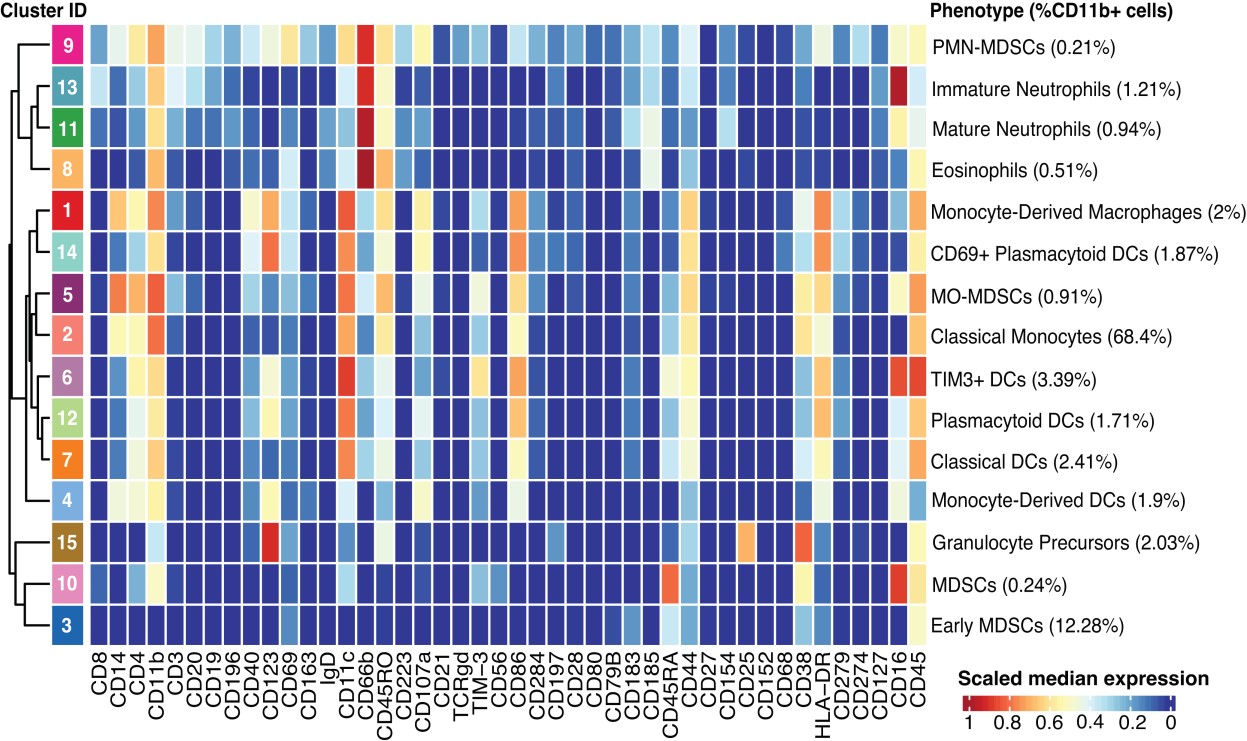


**Supp. Fig. 6. Lineage heatmap of CD11b+ myeloid cells.** CD11b+ cell clusters from Supp. Fig. 1 were combined and re-clustered based on relative expression of canonical myeloid lineage markers (Table 2). Phenotypes for each cluster were inferred using expression of lineage markers and additional functional markers.


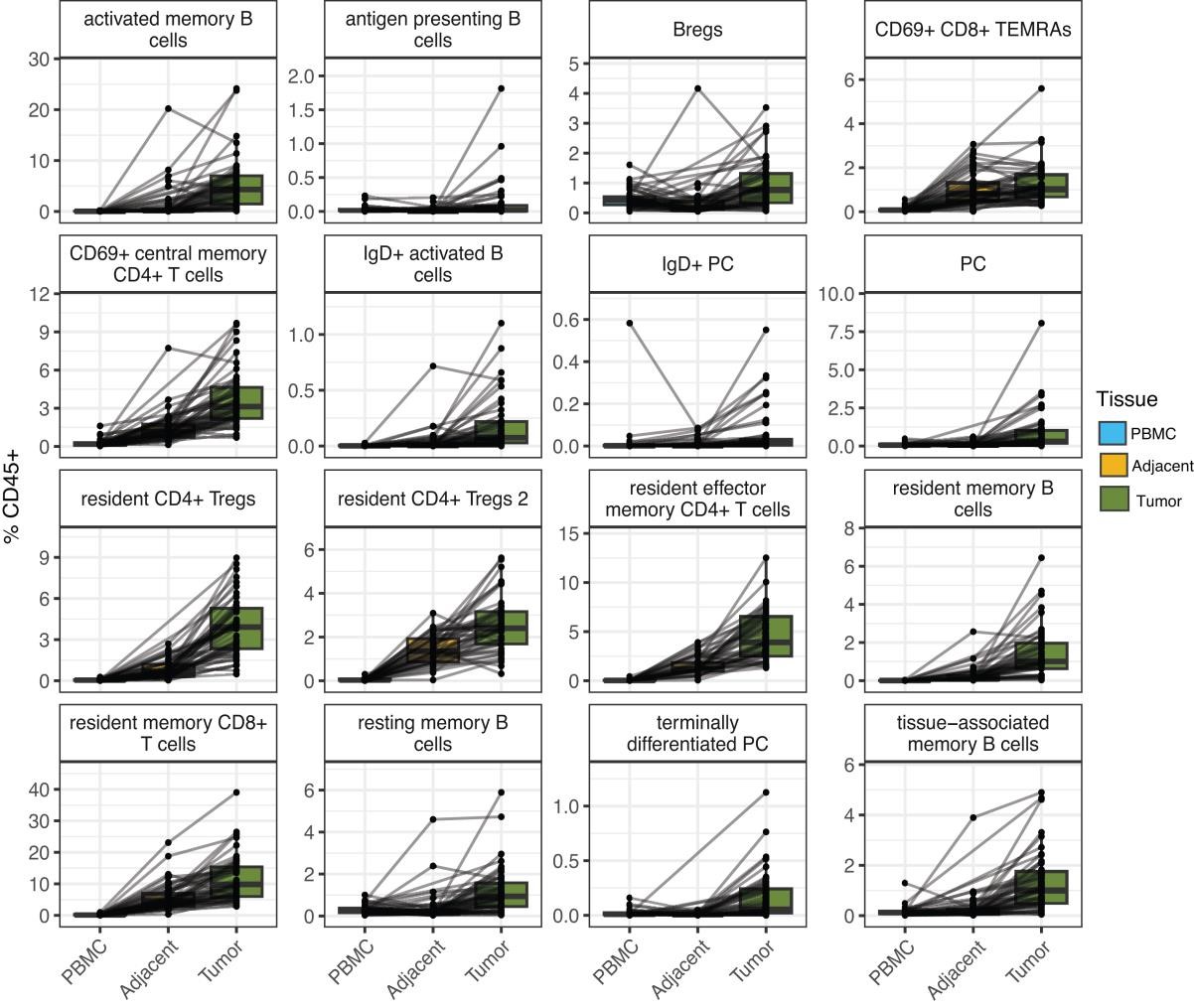


**Supp. Fig. 7. Immune cell subsets enriched in tumor compared to PBMC and adjacent tissues.** Boxplots representing the 16 immune cell subsets that are uniquely elevated in tumor as determined by paired t-tests represented in Fig. 1 B & C.


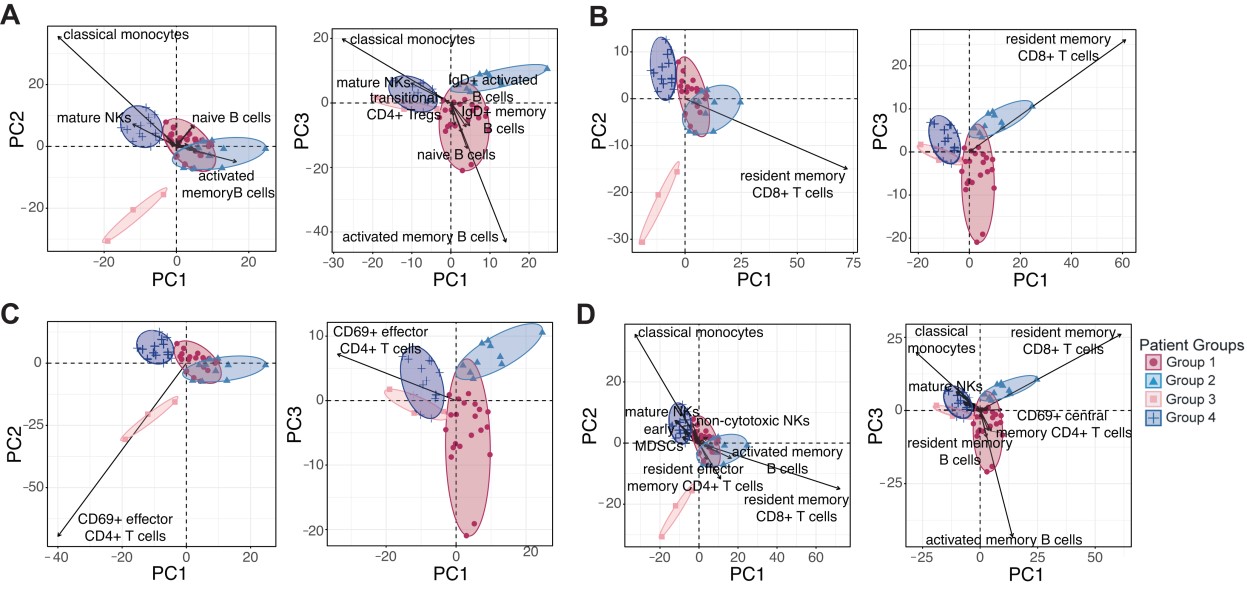


**Supp. Fig. 8. PCA loadings of enriched and depleted immune cell subsets across patient groups.** These plots correspond to the PCA plots in Figure 2 displaying the immune cell subsets identified as significantly enriched or depleted for each patient group by linear regression. Immune cell names are shown on the loadings to visualize how these subsets contribute to group-level separation in PCA space. **(A–D)** PCA loadings for Groups 1–4 respectively, with loadings shown for PC1 vs. PC2 (left) and PC1 vs. PC3 (right).


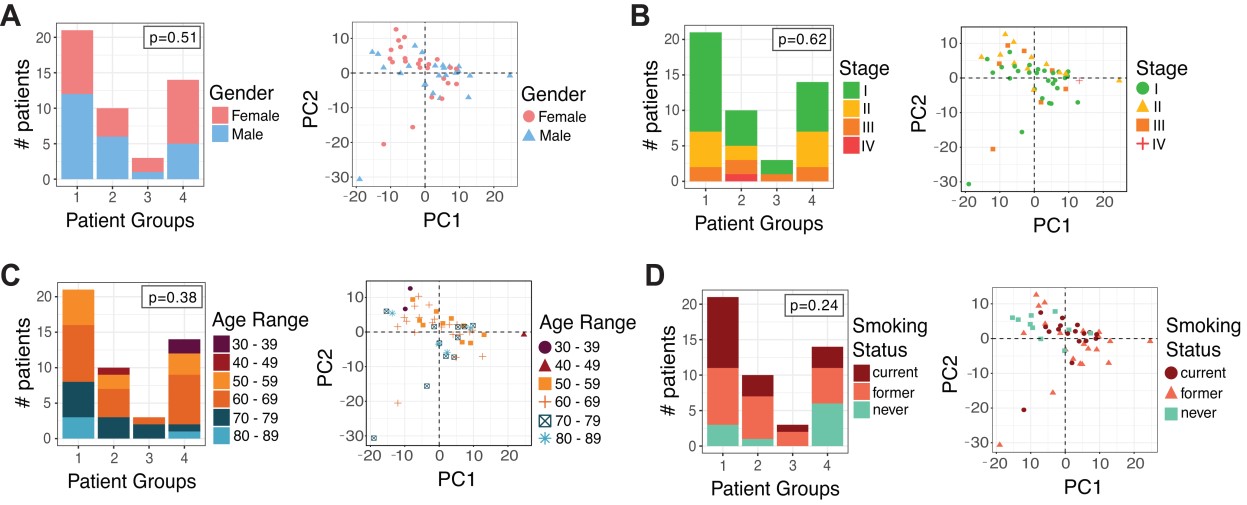


**Supp. Fig. 9. Tumor immune groups are independent of clinical covariates.** Association analyses between patient group assignments (Group 1 n=21, Group 2 n=10, Group 3 n=3, Group 4 n=14) and key clinical variables including tumor stage, biological sex, age, and smoking status. Each variable is represented by bar plots across per patient group (left) and patients are colored accordingly on representative PCA from Fig. 2A (right). (**A**) Distribution of tumor stages (I–IV) across each patient group. **(B)** Proportion of male and female patients in each group. **(C)** Patient age (years at time of diagnosis) plotted by patient group. **(D)** Patient smoking status (at time of diagnosis) across each patient group.

**Supp. Fig. 10: Significant TI immune cell subset proportions.** Boxplots of all 35 significantly enriched or depleted TI immune cell subset proportions represented in Fig. 2C across patient groups (Group 1 n=21, Group 2 n=10, Group 3 n=3, Group 4 n=14).


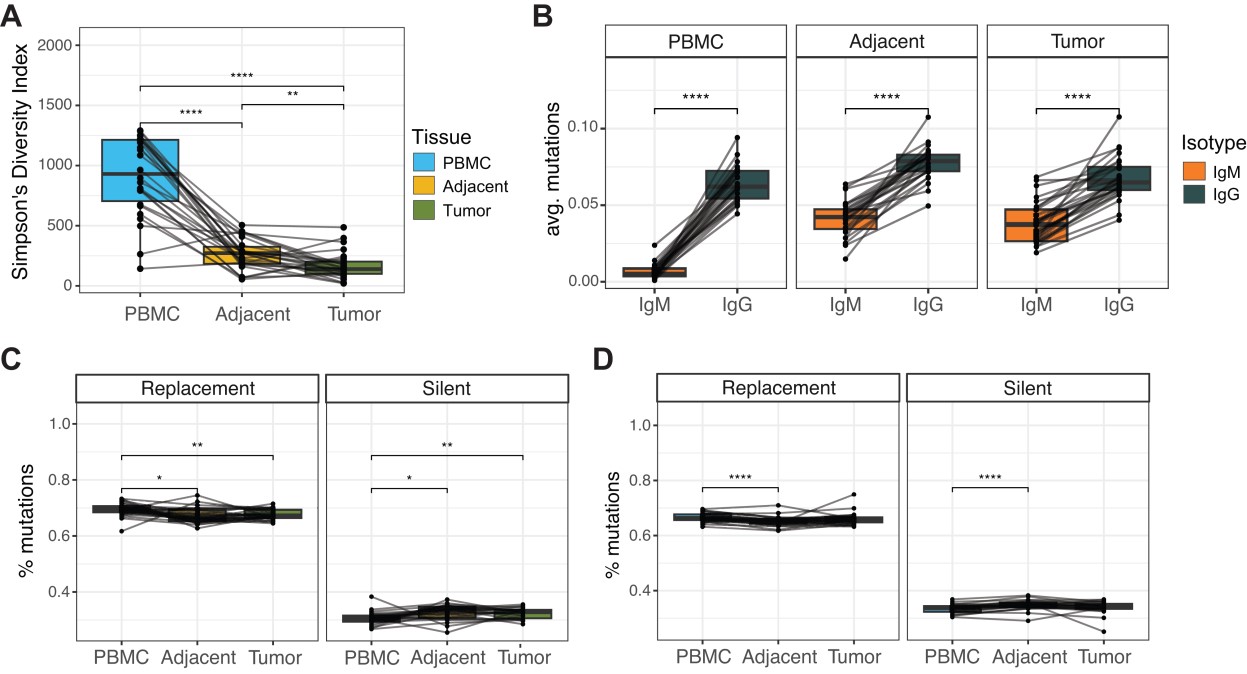


**Supp. Fig. 11: Diversity and SHM patterns across LUAD tissues. (A)** Simpson’s diversity index of combined IgM and IgG repertoires compared across tissues using paired t-tests (n=26). **(B)** Average somatic hypermutation frequency in each tissue compared between matched isotypes using paired t-tests. **(C)** Frequency of replacement (R; left) and silent (S; right) mutations in IgM sequences compared between matched tissues. **(D)** Frequency of replacement (R; left) and silent (S; right) mutations in IgG sequences compared between matched tissues.


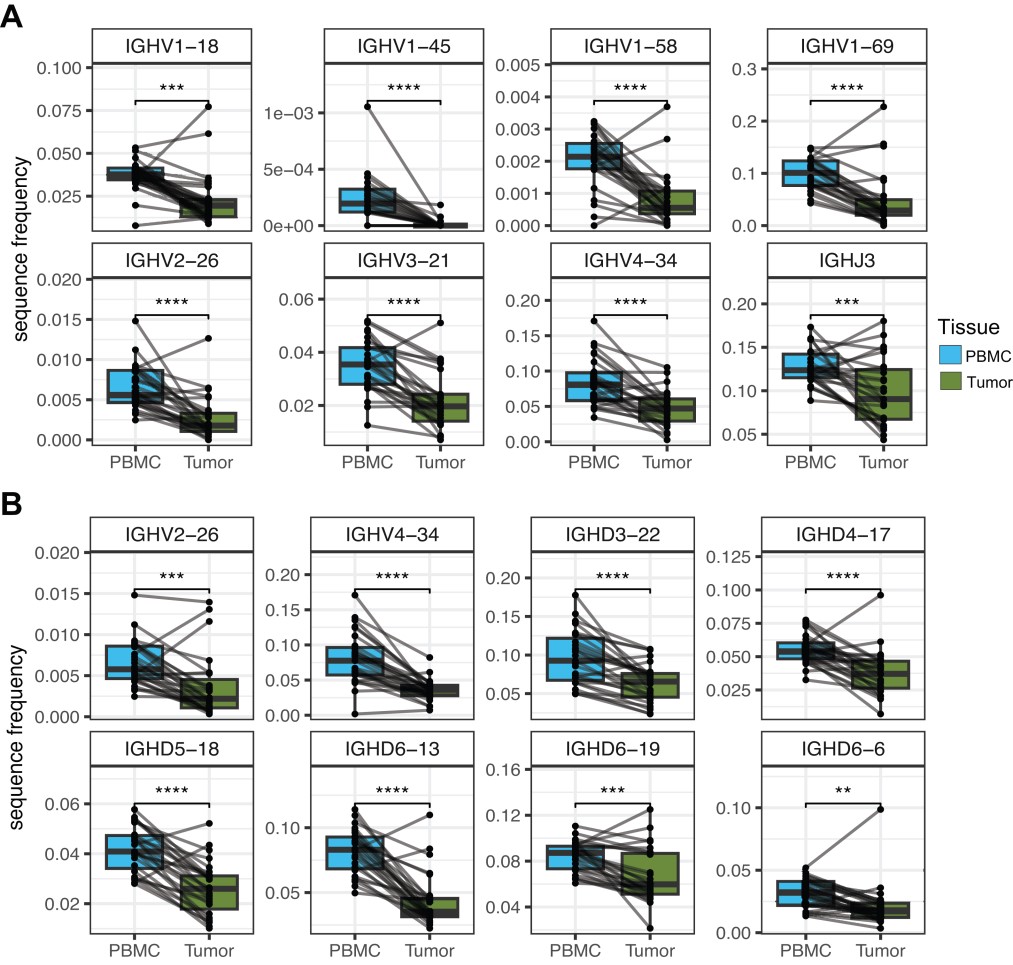


**Supp. Fig. 12: V, D, and J gene segments depleted in tumor IgM and IgG compared to circulating repertoire. (A)** IGHV and IGHJ genes significantly depleted in tumor IgM compared to PBMC IgM (paired t-test p<0.05; n=26). **(B)** IGHV and IGHD genes significantly depleted in tumor IgG compared to PBMC IgM (paired t-test p<0.05, n=27).


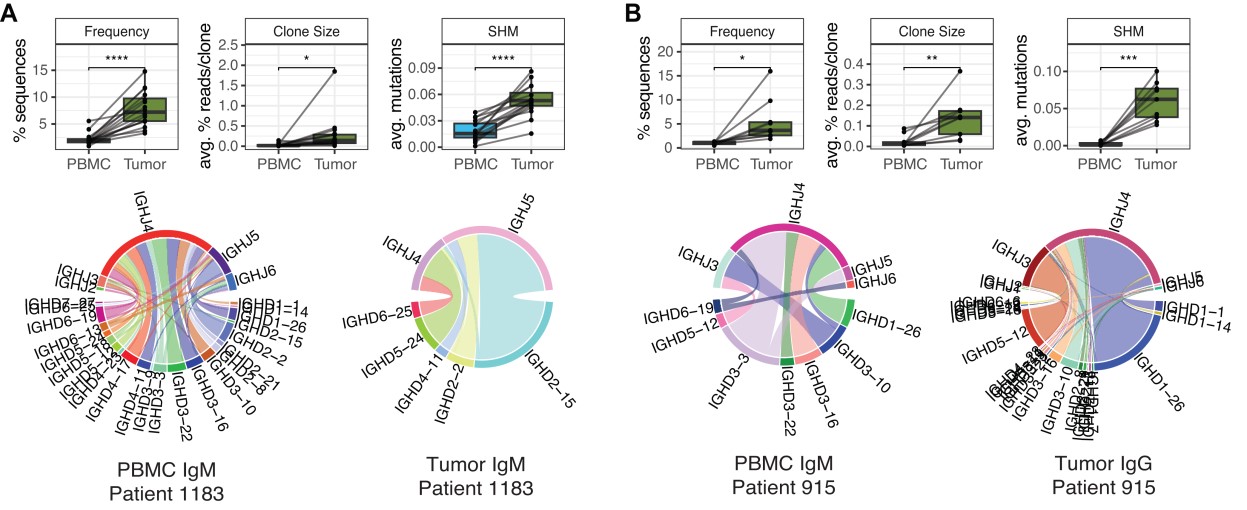


**Supp. Fig. 13: Second most common tumor-enriched IGHV genes.** Panels show IgM enriched gene IGHV3-74 (n=16) **(A)** IgG-enriched gene IGHV1-24 (n=9) **(B)**. Each panel includes relative usage of the IGHV gene in PBMC IgM vs. tumor **(top left)**, distribution of clone sizes **(top center)**, SHM frequency **(top right)**, and IGHD/IGHJ gene pairings in IgM PBMC **(bottom left)** and tumor **(bottom right)** from a representative patient per enriched gene.

**Supplementary Figure 14. IGHG Allelic Variation in LUAD patients.** (**A**) Tile plot of IGHG subisotype alleles across patient groups. Allele name abbreviations and sequences are defined in Supplementary Table 4. (**B**) Heatmap showing Patient 915 IGHJ6 allele frequencies linked to IGHG1 and IGHG4 alleles to resolve haplotypes.

**
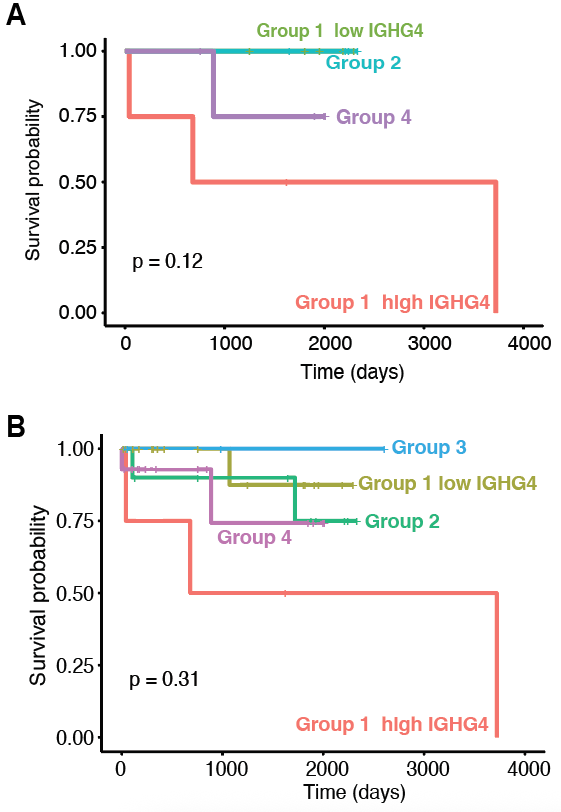
**

**Supplementary Figure 15.** Kaplan-Meier overall survival of IGHG4-high Group 1 patients (n = 4) compared with other patients used in FLAIRR-seq (**A**; Group 1 low IGHG4, n=5; Group 2, n=4; Group 4, n=5) and all other patients (**B**; Group 1 IGHG4-low, n = 17; Group 2, n = 10; Group 3, n = 3; Group 4, n = 14). Global log-rank test used for significance.

# Supp. Table 1. CyTOF antibody staining panel

| **Tag** | **Target** | **Clone** | **Company** | **Details** | **Catalog #** |
| --- | --- | --- | --- | --- | --- |
| 89Y | CD45 | HI30 | Fluidigm | Anti-Human CD45  (HI30)-89Y—100  Tests, 3089003B | 3089003B |
| 106Cd | CD8 | RPA-T8 | Biolegend-Custom |  |  |
| 110Cd | CD14 | M5E2 | Biolegend- Custom |  |  |
| 111Cd | CD4 | RPA-T4 | Biolegend-Custom |  |  |
| 112Cd | CD11b | IRCF44 | Biolegend-Custom |  |  |
| 113Cd | CD3 | UCHT1 | Biolegend-Custom |  |  |
| 114Cd | CD20 | 2H7 | Biolegend-Custom |  |  |
| 116Cd | CD19 | HIB19 | Biolegend-Custom |  |  |
| 141Pr | CD196/CCR6 | G034E4 | Fluidigm | Anti-Human  CD196/CCR6  (G034E3)-141Pr—  50 Tests,  3141003A | 3141003A |
| 142Nd | CD40 | 5C3 | Fluidigm | Anti-Human CD40  (5C3)-142Nd—  100 Tests,  3142010B | 3142010B |
| 143Nd | CD123 | 6H6 | Fluidigm | Anti-Human  CD123/IL-3R  (6H6)-143Nd—  100 Tests,  3143014B | 3143014B |
| 144Nd | CD69 | FN50 | Fluidigm | Anti-Human CD69  (FN50)-144Nd—  100 Tests,  3144018B | 3144018B |
| 145Nd | CD163 | GHI/61 | Fluidigm | Anti-Human  CD163 (GHI/61)-  145ND—100  Tests, 3145010B | 3145010B |
| 146Nd | IgD | IA6-2 | Fluidigm | Anti-Human IgD  (IA6-2)-146Nd— 100 Tests,  3146005B | 3146005B |
| 147Sm | CD11c | Bu15 | Fluidigm | Anti-Human  CD11c (Bu15)-  147Sm—100  Tests, 3147008B | 3147008B |
| 148Nd | CD66b | G10F5 | Biolegend-Custom |  |  |
| 149Sm | CD45RO | UCHL1 | Fluidigm | Anti-Human  CD45RO  (UCHL1)-  149Sm—100  Tests, 3149001B | 3149001B |

| 150Nd | LAG-3 | 11C3C65 | Fluidigm | Anti-Human  CD223/LAG-3  (11C3C65)150Nd—100  Tests, 3150030B | 3150030B |
| --- | --- | --- | --- | --- | --- |
| 151Eu | LAMP1 | H4A3 | Fluidigm | Anti-Human  CD107a/LAMP1  (H4A3)-151Eu—  100 Tests,  3151002B | 3151002B |
| 152Sm | CD21 | BL13 | Fluidigm | Anti-Human CD21  (BL13)-152Sm—  100 Tests,  3152010B | 3152010B |
| 153Eu | γδTCR | B1 | Biolegend-Custom |  |  |
| 154Sm | TIM-3 | F38-2E2 | Fluidigm | Anti-Human TIM-3  (F38-2E2)154Sm—100  Tests, 3154010B | 3154010B |
| 155Gd | CD56 | HCD56 | Fluidigm | Anti-Human CD56  (B159)-155Gd—  100 Tests,  3155008B | 3155008B |
| 156Gd | CD86 | IT2.2 | Fluidigm | Anti-Human  CD86/B7.2  (IT2.2)-156Gd— 100 Tests,  3156008B | 3156008B |
| 158Gd | TLR4 | HTA125 | Fluidigm | Anti-Human  CD284/TLR4  (HTA125)158Gd—100  Tests, 3158024B | 3158024B |
| 159Tb | CD197/CCR7 | G043H7 | Fluidigm | Anti-Human  CD197/CCR7  (G043H7)159Tb—50 Tests,  3159003A | 3159003A |
| 160Gd | CD28 | CD28.2 | Fluidigm | Anti-Human CD28 (CD28.2)-160Gd—  100 Tests,  3160003B | 3160003B |
| 161Dy | CD80 | 2D10.4 | Fluidigm | Anti-Human  CD80/B7-1  (2D10.4)-161Dy— 100 Tests,  3161023B | 3161023B |
| 162Dy | CD79b | CB3-1 | Fluidigm | Anti-Human  CD79B (CB3-1)- | 3162008B |

|  |  |  |  | 162Dy—100 Tests, 3162008B |  |
| --- | --- | --- | --- | --- | --- |
| 163Dy | CXCR3/CD183 | G025H7 | Fluidigm | Anti-Human  CD183/CXCR3  (G025H7)163Dy—100  Tests, 3163004B | 3163004B |
| 164Dy | CXCR5/CD185 | RF8B2 | Fluidigm | Anti-Human  CD185/CXCR5  (RF8B2)-164Dy—  100 Tests,  3164029B | 3164029B |
| 165Ho | CD45RA | HI100 | Biolegend-Custom |  |  |
| 166Er | CD44 | BJ18 | Fluidigm | Anti-Human CD44  (BJ18)-166Er— 100 Tests,  3166001B | 3166001B |
| 167Er | CD27 | L128 | Fluidigm | Anti-Human CD27  (L128)-167Er— 100 Tests,  3167006B | 3167006B |
| 168Er | CD40L | 24-31 | Fluidigm | Anti-Human  CD154/CD40L  (24-31)-168Er— 100 Tests,  3168006B | 3168006B |
| 169Tm | CD25 | 2A3 | Fluidigm | Anti-Human CD25  (2A3)-169Tm—  100 Tests,  3169003B | 3169003B |
| 170Er | CTLA-4 | 14D3 | Fluidigm | Anti-Human  CD152/CTLA-4  (14D3)-170Er— 100 Tests,  3170005B | 3170005B |
| 171Yb | CD68 | Y1/82A | Fluidigm | Anti-Human CD68  (Y1/82A)-171Yb— 100 Tests,  3171011B | 3171011B |
| 172Yb | CD38 | HIT2 | Fluidigm | Anti-Human CD38  (HIT2)-172Yb— 100 Tests,  3172007B | 3172007B |
| 173Yb | HLA-Dr | L243 | Fluidigm | Anti-Human HLADR (L243)173Yb—100  Tests, 3173005B | 3173005B |
| 174Yb | CD279/PD-1 | EH12.2H7 | Fluidigm | Anti-Human  CD279/PD-1  (EH12.2H7)- | 3174020B |
|  |  |  |  | 174Yb—100 Tests, 3174020B |  |
| 175Lu | CD274/PD-L1 | 29E.2A3 | Fluidigm | Anti-Human  CD274/PDL1  (29E.2A3)175Lu—100  Tests, 3175017B | 3175017B |
| 176Yb | CD127/IL-7R | A019D5 | Fluidigm | Anti-Human  CD127/IL-7Ra  (A019D5)176Yb—100  Tests, 3176004B | 3176004B |
| 209Bi | CD16 | 3G8 | Fluidigm | Anti-Human CD16 (3G8)-209Bi—100  Tests, 3209002B | 3209002B |

# Supp. Table 2. FLAIRR-seq primers and barcodes

| **Primer**  **Name** | **Barcode 5'→3'** | **Target 5'→3'** | **Final Sequence 5'→3'** |
| --- | --- | --- | --- |
| IgG_CH  3_bc100  1 | CACATATCAGAGTGCG | CATGCATCA  CGGAGCATG  AG | CACATATCAGAGTGCGC  ATGCATCACGGAGCATG  AG |
| IgG_CH  3_bc100  2 | ACACACAGACTGTGAG | CATGCATCA  CGGAGCATG  AG | ACACACAGACTGTGAGC  ATGCATCACGGAGCATG  AG |
| IgG_CH  3_bc100  3 | ACACATCTCGTGAGAG | CATGCATCA  CGGAGCATG  AG | ACACATCTCGTGAGAGC  ATGCATCACGGAGCATG  AG |
| IgG_CH  3_bc100  4 | CACGCACACACGCGCG | CATGCATCA  CGGAGCATG  AG | CACGCACACACGCGCGC  ATGCATCACGGAGCATG  AG |
| IgG_CH  3_bc100  5 | CACTCGACTCTCGCGT | CATGCATCA  CGGAGCATG  AG | CACTCGACTCTCGCGTC  ATGCATCACGGAGCATG  AG |
| IgG_CH  3_bc100  6 | CATATATATCAGCTGT | CATGCATCA  CGGAGCATG  AG | CATATATATCAGCTGTCA  TGCATCACGGAGCATGA  G |
| IgG_CH  3_bc100  8 | ACAGTCGAGCGCTGCG | CATGCATCA  CGGAGCATG  AG | ACAGTCGAGCGCTGCGC  ATGCATCACGGAGCATG  AG |
| IgG_CH  3_bc101  2 | ACACTAGATCGCGTGT | CATGCATCA  CGGAGCATG  AG | ACACTAGATCGCGTGTC  ATGCATCACGGAGCATG  AG |
| IgM_CH  4_bc100  1 | CACATATCAGAGTGCG | GTCTCCCCC  GTGTTCCATT  C | CACATATCAGAGTGCGG  TCTCCCCCGTGTTCCATT  C |
| IgM_CH  4_bc100  2 | ACACACAGACTGTGAG | GTCTCCCCC  GTGTTCCATT  C | ACACACAGACTGTGAGG  TCTCCCCCGTGTTCCATT  C |
| IgM_CH  4_bc100  3 | ACACATCTCGTGAGAG | GTCTCCCCC  GTGTTCCATT  C | ACACATCTCGTGAGAGG  TCTCCCCCGTGTTCCATT  C |
| IgM_CH  4_bc100  4 | CACGCACACACGCGCG | GTCTCCCCC  GTGTTCCATT  C | CACGCACACACGCGCG  GTCTCCCCCGTGTTCCA  TTC |
| IgM_CH  4_bc100  5 | CACTCGACTCTCGCGT | GTCTCCCCC  GTGTTCCATT  C | CACTCGACTCTCGCGTG  TCTCCCCCGTGTTCCATT  C |
| IgM_CH  4_bc100  6 | CATATATATCAGCTGT | GTCTCCCCC  GTGTTCCATT  C | CATATATATCAGCTGTGT  CTCCCCCGTGTTCCATT  C |
| IgM_CH  4_bc100  8 | ACAGTCGAGCGCTGCG | GTCTCCCCC  GTGTTCCATT  C | ACAGTCGAGCGCTGCGG  TCTCCCCCGTGTTCCATT  C |
| IgM_CH  4_bc101  2 | ACACTAGATCGCGTGT | GTCTCCCCC  GTGTTCCATT  C | ACACTAGATCGCGTGTG  TCTCCCCCGTGTTCCATT  C |
| TSO_U  MI | 5’-  AAGCAGUGGTAUCAACGCAGA GUNNNNUNNNNUNNNNUCTTrG rGrG -3’ |  |  |

# Supp. Table 3. Summary of patient samples included in each experimental method by patient group. The number of patients per group (Groups 1–4) used for each assay and tissue type, the number of patients not included in CyTOF, and the total number of patients represented in each method. “AIRR” refers to adaptive immune receptor repertoire sequencing; “FLAIRR” refers to full-length adaptive immune receptor repertoire sequencing. *Group 4 includes one patient tumor IgG used in FLAIRR-seq but no samples for that patient were used in AIRR-seq.

| **Method** | **Group 1**  **(# patients)** | **Group 2**  **(# patients)** | **Group 3**  **(# patients)** | **Group 4***  **(# patients)** | **patients**  **not used in CyTOF** | **Total**  **(# patients)** |
| --- | --- | --- | --- | --- | --- | --- |
| **Confocal**  **(Tumor &**  **Adjacent)** | 0 | 0 | 0 | 0 | 1 | 1 |
| **Flow**  **Cytometry**  **(Tumor &**  **Adjacent)** | 5 | 3 | 0 | 0 | 4 | 12 |
| **CyTOF Tumor** | 21 | 10 | 3 | 14 | 0 | 48 |
| **CyTOF PBMC** | 19 | 9 | 3 | 13 | 0 | 44 |
| **CyTOF**  **Adj** | 18 | 10 | 2 | 12 | 0 | 42 |
| **AIRR**  **Tumor IgG** | 12 | 7 | 1 | 4* | 5 | 29 |
| **AIRR**  **Tumor**  **IgM** | 12 | 7 | 1 | 3 | 5 | 28 |
| **AIRR**  **PBMC IgG** | 11 | 7 | 1 | 3 | 5 | 27 |
| **AIRR**  **PBMC IgM** | 11 | 7 | 1 | 3 | 5 | 27 |
| **AIRR Adj. IgG** | 11 | 7 | 1 | 2 | 5 | 26 |
| **AIRR Adj. IgM** | 11 | 7 | 1 | 2 | 5 | 26 |
| **FLAIRR** | 9 | 4 | 0 | 5* | 0 | 18 |

**Supp. Table 4. IGHC allele designations and abbreviations.** Most allele calls did not exactly match existing reference databases; putative novel alleles are labeled by the closest known allele with the observed sequence differences indicated.

| Abbreviated name | Allele |
| --- | --- |
| IGHG1*03_var1 | IGHG1*03_g1172a |
| IGHG1*03_var2 | IGHG1*03_g1172a_like |
| IGHG1*05 | IGHG1*05_a959g_a1192g |
| IGHG1*08 | IGHG1*08_g1190a |
| IGHG1*15_var1 | IGHG1*15_c940g |
| IGHG1*15_var2 | IGHG1*15_c940g_like |
| IGHG1*15_var3 | IGHG1*15_g119a |
| IGHG2*02 | IGHG2*02_c1177t |
| IGHG2*03_var1 | IGHG2*03_c1177a_like |
| IGHG2*03_var2 | IGHG2*03_t923c |
| IGHG2*03_var3 | IGHG2*03_t923c_like |
| IGHG2*06 | IGHG2*06 |
| IGHG2*18 | IGHG2*18_a818g_c1039g |
| IGHG3*03_var1 | IGHG3*03_a374g_g419a_a426c_a631t_g933a_g970a_a974c_g999c_t1007c_g1008a_g1162c |
| IGHG3*03_var2 | IGHG3*03_fe8d |
| IGHG3*03_var3 | IGHG3*03_g1162c |
| IGHG3*10 | IGHG3*10 |
| IGHG3*11 | IGHG3*11 |
| IGHG3*11_var1 | IGHG3*11_c769g |
| IGHG3*11_var2 | IGHG3*11_like |
| IGHG3*14_var1 | IGHG3*14_a804g |
| IGHG3*14_var2 | IGHG3*14_g1336- |
| IGHG3*21 | IGHG3*21 |
| IGHG4*01 | IGHG4*01 |
| IGHG4*01_var1 | IGHG4*01_a703c |
| IGHG4*01_var2 | IGHG4*01_c20g_c56t_c188t_a314g_g338a |
| IGHG4*01_var3 | IGHG4*01_like |
| IGHG4*01_var4 | IGHG4*01_t2c_c68g_c251t |
| IGHG4*04 | IGHG4*04_like |
| IGHG4*08 | IGHG4*08_conv_6/IGHG4D*01_a314g_g958a |
| IGHG4*09_var1 | IGHG4*09_a314g_a865g_c869a |
| IGHG4*09_var2 | IGHG4*09_a865g_c869a_g1072a |
| IGHG4*09_var3 | IGHG4*09_a865g_c869a_g1072a_like |
